# Mitochondrial Fission Factor Is a Novel Interacting Protein of the Critical B Cell Survival Regulator TRAF3 in B Lymphocytes

**DOI:** 10.3389/fimmu.2021.670338

**Published:** 2021-10-20

**Authors:** Yingying Liu, Samantha Gokhale, Jaeyong Jung, Sining Zhu, Chang Luo, Debanjan Saha, Jessie Yanxiang Guo, Huaye Zhang, Saw Kyin, Wei-Xing Zong, Eileen White, Ping Xie

**Affiliations:** ^1^ Department of Cell Biology and Neuroscience, Rutgers University, Piscataway, NJ, United States; ^2^ Graduate Program in Cellular and Molecular Pharmacology, Rutgers University, Piscataway, NJ, United States; ^3^ Rutgers Cancer Institute of New Jersey, New Brunswick, NJ, United States; ^4^ Department of Medicine, Rutgers Robert Wood Johnson Medical School, New Brunswick, NJ, United States; ^5^ Department of Chemical Biology, Rutgers Ernest Mario School of Pharmacy, Piscataway, NJ, United States; ^6^ Department of Neuroscience and Cell Biology, Rutgers Robert Wood Johnson Medical School, Piscataway, NJ, United States; ^7^ Department of Molecular Biology, Princeton University, Princeton, NJ, United States; ^8^ Department of Molecular Biology and Biochemistry, Rutgers University, Piscataway, NJ, United States

**Keywords:** TRAF3, MFF, mitochondria, apoptosis, B lymphocytes, B cell malignancies

## Abstract

Proteins controlling mitochondrial fission have been recognized as essential regulators of mitochondrial functions, mitochondrial quality control and cell apoptosis. In the present study, we identified the critical B cell survival regulator TRAF3 as a novel binding partner of the key mitochondrial fission factor, MFF, in B lymphocytes. Elicited by our unexpected finding that the majority of cytoplasmic TRAF3 proteins were localized at the mitochondria in resting splenic B cells after *ex vivo* culture for 2 days, we found that TRAF3 specifically interacted with MFF as demonstrated by co-immunoprecipitation and GST pull-down assays. We further found that in the absence of stimulation, increased protein levels of mitochondrial TRAF3 were associated with altered mitochondrial morphology, decreased mitochondrial respiration, increased mitochondrial ROS production and membrane permeabilization, which eventually culminated in mitochondria-dependent apoptosis in resting B cells. Loss of TRAF3 had the opposite effects on the morphology and function of mitochondria as well as mitochondria-dependent apoptosis in resting B cells. Interestingly, co-expression of TRAF3 and MFF resulted in decreased phosphorylation and ubiquitination of MFF as well as decreased ubiquitination of TRAF3. Moreover, lentivirus-mediated overexpression of MFF restored mitochondria-dependent apoptosis in TRAF3-deficient malignant B cells. Taken together, our findings provide novel insights into the apoptosis-inducing mechanisms of TRAF3 in B cells: as a result of survival factor deprivation or under other types of stress, TRAF3 is mobilized to the mitochondria through its interaction with MFF, where it triggers mitochondria-dependent apoptosis. This new role of TRAF3 in controlling mitochondrial homeostasis might have key implications in TRAF3-mediated regulation of B cell transformation in different cellular contexts. Our findings also suggest that mitochondrial fission is an actionable therapeutic target in human B cell malignancies, including those with *TRAF3* deletion or relevant mutations.

## Highlights

- Cytoplasmic TRAF3 is mainly localized at mitochondria and interacts with MFF in B lymphocytes after 2 days in culture.
- TRAF3 regulates the phosphorylation and ubiquitination of MFF, mitochondrial morphology, respiration and ROS production to induce apoptosis.
- Overexpression of MFF restores mitochondria-dependent apoptosis in TRAF3-deficient malignant B cells.

## Introduction

B cell malignancies comprise the majority of human blood cancers and represent the most common types of lymphoid tumors (1–3). One essential pathogenic mechanism underlying B cell malignant transformation is the dysregulation of the survival and apoptosis pathways, including the B cell receptor (BCR)-Btk, NF-κB1/2-Bcl-2, PI-3K-Akt-mTOR, c-Myc-ERK and Jak-STAT signaling axes (4–12). Recurrent genetic alterations that lead to the activation/elevation of the survival signaling pathways or inhibition/reduction of the apoptosis signaling pathways are frequently detected in various B cell malignancies. Typical examples are gene amplifications, chromosomal translocations or activating mutations that result in increased expression levels or constitutive activation of critical pro-survival and anti-apoptotic proteins such as NIK, c-Rel, c-Myc, Bcl-2, Btk, and p110^δ^ of PI-3K (4–12). Alternative examples include gene deletions, chromosomal loss or inactivating mutations that cause decreased expression levels or impaired activities of inhibitors of the survival pathways and pro-apoptotic proteins such as IκBα, A20, PTEN, DUSP2, Fas and Bim (4–12). Such deregulation of the survival and apoptosis pathways not only contributes to the pathogenesis but also mediates resistance to various therapies in patients with B cell malignancies (4–11). Therefore, therapeutic strategies and drugs aimed at targeting the survival pathways or restoring the apoptosis pathways are being developed and tested in clinical trials as effective treatments for B cell malignancies, and particularly as useful adjuvants to overcome resistance to other therapies (4–11). Better understanding of the regulatory mechanisms of B cell survival and apoptosis will facilitate the development and improvement of these therapeutic strategies.

One of the most frequently deleted or mutated survival regulator in human B cell malignancies is TRAF3 (13, 14), a cytoplasmic adaptor protein that has been identified as a critical regulator of cell survival in mature B lymphocytes (13, 15–19). Deletions and inactivating mutations of the *TRAF3* gene have been documented in almost all malignancies of mature B cells, including multiple myeloma (MM), diffuse large B-cell lymphoma (DLBCL), B-cell chronic lymphocytic leukemia (B-CLL), gastric and splenic marginal zone lymphoma (MZL), Hodgkin lymphoma (HL) and Waldenstrom’s macroglobulinemia (WM) (13, 14). Specific deletion of the *Traf3* gene from B lymphocytes in mice results in severe peripheral B cell hyperplasia due to the prolonged survival of mature B cells independent of the principle B cell survival factor BAFF (15, 16), which eventually leads to spontaneous development of splenic MZL and B1 lymphomas (19). Mechanistically, the TRAF3-TRAF2-cIAP1/2 complex constitutively targets the NF-κB-inducing kinase (NIK) for K48-linked polyubiquitination and proteasome-dependent degradation (20–23). Ablation of TRAF3 as well as TRAF2 or cIAP1/2 all results in constitutive NF-κB2 activation and prolonged survival of mature B lymphocytes (15, 16, 24). Furthermore, we recently elucidated that TRAF3 also regulates the choline kinase α (Chkα)-mediated phosphocholine and phosphatidylcholine (PC)/phosphatidylethanolamine (PE) biosynthesis pathways to control the survival of mature B cells (25). Paradoxically, transgenic overexpression of *TRAF3* in B cells promotes B cell differentiation and results in plasmacytosis and autoimmunity in mice (26), while double transgenic overexpression of both *TRAF3* and *BCL-2* in B cells leads to the development of multiple classes of mature non-Hodgkin B cell lymphomas in mice (27). Detailed mechanisms underlying such seemingly opposite roles of TRAF3 in B cell tumorigenesis were unknown, but all the above findings consistently indicate that TRAF3 is a master regulator of B cell survival and function.

It is known that in the absence of stimulation, TRAF3 proteins are distributed in the cytoplasm and nucleus (28, 29). In the present study, we were interested to determine whether TRAF3 proteins are evenly distributed within the cytoplasm in resting B cells. We obtained an unexpected finding that the majority of cytoplasmic TRAF3 proteins were localized at the mitochondria in resting splenic B cells after 2 days in culture. Given the central importance of mitochondria in regulating cell survival and apoptosis (30–32), our unexpected finding of the mitochondrial localization of TRAF3 proteins led us to test a novel hypothesis that in addition to the previously elucidated TRAF3-NIK-NF-κB2 and Chkα-phosphocholine-PC/PE pathways, TRAF3 can directly regulate the physiology of mitochondria to control B cell apoptosis. Our results described in this paper provide interesting evidence to support this hypothesis. We identified mitochondrial fission factor (MFF), a mitochondrial outer membrane (MOM) protein, as a novel TRAF3-interacting protein in B cells. We demonstrated that TRAF3 inhibited the posttranslational modifications of MFF, regulated mitochondrial morphology and function, and promoted mitochondria-dependent apoptosis in B cells. Our findings thus shed new light into the complex apoptosis-inducing mechanisms of TRAF3 in B cells. Furthermore, our study discovered additional targetable points, MFF and mitochondrial fission, for the treatment of human B cell malignancies, especially those involving *TRAF3* deletion or relevant mutations.

## Materials and Methods

### Mice and Cell Lines

*Traf3* ^flox/flox^CD19^+/Cre^ (B-*Traf3* ^-/-^) and *Traf3* ^flox/flox^ (littermate control, LMC) mice were generated as previously described (15). All experimental mice for this study were produced by breeding *Traf3* ^flox/flox^ mice with *Traf3* ^flox/flox^CD19^+/Cre^ mice. All mice were kept in specific pathogen-free conditions in the Animal Facility at Rutgers University, and were used in accordance with NIH guidelines and under an animal protocol (Protocol # 08-048) approved by the Animal Care and Use Committee of Rutgers University. Equal numbers of male and female mice were used in this study.

The human multiple myeloma (MM) cell line 8226, which contains bi-allelic *TRAF3* deletions, was kindly provided by Dr. Leif Bergsagel (Mayo Clinic, Scottsdale, AZ) and was cultured as previously described (33). The human embryonic kidney 293T cell line was purchased from American Type Culture Collection (ATCC, Manassas, VA) and was cultured according to the manufacturer’s protocol.

### Reagents and Antibodies

Mitoprobe JC-1 Assay Kit, MitoSOX Red, and tissue culture supplements including stock solutions of sodium pyruvate, L-glutamine, non-essential amino acids and HEPES (pH 7.55) were from Invitrogen (Carlsbad, CA). Fluorochrome-labeled antibodies (Abs) against Annexin V and mouse Thy1.1 were purchased from BioLegend (San Diego, CA). Propidium iodide (PI), MG-132 and the sarkosyl detergent (N-laurylsarcosine sodium) were purchased from Sigma-Aldrich Corp. (St. Louis, MO). Mitochondria Isolation Kit was purchased from ThermoFisher (Waltham, MA). Recombinant BAFF was from PeproTech (Rocky Hill, NJ) and agonistic anti-CD40 (HM40-3) was purchased from eBioscience (San Diego, CA). The TnT^®^ Quick T7 Coupled Transcription/Translation System was from Promega (Madison, WI). Seahorse XF Cell Mito Stress Test Kit was obtained from Agilent Technologies (Lexington, MA). EDTA-free Protease Inhibitor Cocktail Tablets were obtained from Roche Diagnostics Corp (Indianapolis, IN). Phosphatase Inhibitor Mini Tablets, the deubiquitinase inhibitor N-ethylmaleimide (NEM), GelCode Blue Stain reagent and Streptavidin-Sepharose beads were purchased from Pierce (Rockford, IL). Glutathione-Sepharose 4B beads were from GE Healthcare (Chicago, IL). Anti-c-Myc Tag (9E10) Affinity Gel was from BioLegend (San Diego, CA). Bradford Assay was purchased from Bio-Rad (Hercules, CA). Polyclonal or monoclonal rabbit Abs against total or phosphorylated MFF, Caspase 9, Caspase 3, Calreticulin, COX IV, YY1, Bcl-xL, Bcl-2, Mcl-1, Bax, ubiquitin (Ub), K48-Ub, K63-Ub and HA tag were from Cell Signaling Technology (Beverly, MA). Polyclonal rabbit Abs to TRAF3 (H122) and Myc tag were from Santa Cruz Biotechnology (Santa Cruz, CA). Mouse monoclonal Abs to SBP tag was purchased from EMD Millipore Corp (Burlington, MA). Anti-β-actin Ab was from Chemicon (Temecula, CA). HRP-labeled secondary Abs were from Jackson ImmunoResearch Laboratories, Inc. (West Grove, PA).

### DNA Constructs

The full-length coding cDNA sequence of human TRAF3 was cloned and the lentiviral expression vector pUB-TRAF3-Thy1.1 was generated as previously described (25). To facilitate co-immunoprecipitation and affinity purification, we engineered an N-terminal FLAG tag or a C-terminal streptavidin-binding peptide-6xHistidine (SBP-6xHis) tag (34) in frame with the TRAF3 coding sequence, respectively. We subsequently generated two lentiviral expression vectors of tagged human TRAF3, including pUB-FLAG-TRAF3 and pUB-TRAF3-SBP-6xHis. For GST pull-down studies, we cloned the human TRAF3 coding sequence into the pGEX vector (provided by Dr. Mike Kiledjian, Rutgers University) (35) and generated the pGEX-GST-TRAF3 plasmid that expresses the GST-TRAF3 fusion protein. We also engineered several deletion mutants of human TRAF3 that lack different structural domains (28) using PCR cloning, including ΔTRAF-C (lacks the TRAF-C domain), ΔTRAF-N&C (lacks the TRAF-N and TRAF-C domains), ΔZnR (lacks the Zinc RING domain) and ΔZnR&F (lacks the Zinc RING domain and all 5 Zinc fingers). The coding cDNA sequences of MFF were cloned from the human MM cell line 8226 cells using reverse transcription PCR with the high fidelity polymerase Pfu UltraII (Agilent, Santa Clara, CA). Primers used for the cloning of human MFF include hMFF isoform 1-F (5’- ATT TAA ATG AGT AAA GGA ACA AGC A -3’) or hMFF isoform 2 or isoform 3-F (5’- GCT GAG ATG GCA GAA ATT AGT CGA ATT -3’) paired with hMFF-R (5’- CTC TAG CGG CGA AAC CAG AGC CA -3’). No PCR products of human MFF isoform 1 were amplified from 8226 cells. PCR products of human MFF isoform 2 or 3 were gel-purified and verified by DNA sequencing. To facilitate immunoprecipitation studies, we engineered an N-terminal Myc tag (34) in frame with the human MFF coding sequences and subsequently cloned them into the expression vector pcDNA3.0. The coding cDNA sequence of each Myc-tagged MFF isoform was also subcloned into the lentiviral expression vector pUB-eGFP-Thy1.1 (36) (generously provided by Dr. Zhibin Chen, the University of Miami, Miami, FL) by replacing the eGFP coding sequence with Myc-MFF. The lentiviral expression vector of HA-tagged ubiquitin LPC-HA-Ub was described previously (37). Each DNA construct was verified by DNA sequencing at GenScript (Piscataway, NJ).

### Splenic B Cell Purification and Culture

Mouse splenic B cells were purified using anti-mouse CD43-coated magnetic beads and a MACS separator (Miltenyi Biotec Inc.) following the manufacturer’s protocols as previously described (15). The purity of the isolated B cell population was monitored by FACS analysis and cell preparations of >98% B220+CD3- purity (**Supplementary Figure 1**) were used for protein preparation and mitochondrial analyses. An aliquot of purified splenic B cells was cultured *ex vivo* in mouse B cell medium (15, 25) for 1 or 2 days before protein preparation and mitochondrial analyses.

### Transduction of Human MM Cells With Lentiviral Expression Vectors

Lentiviruses of pUB-TRAF3-SBP-6xHis, pUB-FLAG-TRAF3, pUB-Myc-MFF, pUB-Myc-MFF3 and an empty vector pUB-Thy1.1 were packaged and lentiviral titers were determined as previously described (33, 38). Human MM 8226 cells were transduced with the packaged lentiviruses at an MOI of 1:5 (cell:virus) in the presence of 8 μg/mL polybrene (33, 38). Transduction efficiency of cells was analyzed at day 3 post transduction using Thy1.1 immunofluorescence staining followed by flow cytometry. Transduced cells were subsequently used for mitochondrial isolation and affinity purification or apoptosis analyses.

### Flow Cytometry

For analysis of apoptosis, cells were stained with annexin V according to the manufacturer’s protocol (Invitrogen) and analyzed by flow cytometry as previously described (19). For cell cycle analysis, cells were fixed with ice-cold 70% ethanol. Cell cycle distribution was subsequently determined by propidium iodide (PI) staining followed by flow cytometry as previously described (39). For the measurement of mitochondrial membrane permeabilization, cells were stained with a MitoProbe JC-1 Assay Kit (Molecular Probes) following the manufacturer’s instructions. Briefly, 2 x 10^6^ cells of each condition were resuspended in 1 ml PBS and incubated for 5 minutes at 37°C. Subsequently, 10 µl of 200 µM JC-1 (final concentration: 2 µM) was added to the cells and incubated at 37°C for 30 minutes. Cells were subsequently washed with 2 ml of PBS, fixed with 1% formaldehyde, and then analyzed by flow cytometry. Listmode data were acquired on a Northern Lights spectral flow cytometer (Cytek, Fremont, CA) or a FACSCalibur (Becton Dickinson, Mountain View, CA). The results were analyzed using the FlowJo software (TreeStar, San Carlos, CA).

### Total Protein Extraction and Immunoblot Analysis

For total protein lysates, cell pellets were lysed in 2X SDS sample buffer (62.5 mM Tris, pH6.8, 1% SDS, 15% glycerol, 2% β-mercaptoethanol and 0.005% bromophenol blue), sonicated for 30 pulses, and then boiled for 10 minutes (40). Proteins were separated by SDS-PAGE and immunoblotted with antibodies to specific proteins as indicated in the figures followed by HRP-conjugated secondary antibodies (goat anti-rabbit or goat anti-mouse IgG). A chemiluminescent substrate (Pierce) was used to detect HRP-labeled Abs on immunoblots. Images of chemiluminescence signals on immunoblots were acquired and quantitated using a low-light imaging system (LAS-4000 mini, FUJIFILM Medical Systems USA, Inc., Stamford, CT) (19, 33).

### Fractionation of Cytosol, Mitochondria and Microsomes (Rich in ER)

For purified mouse splenic B cells (8 x 10^7^ cells/condition), mitochondria were fractionated from cells using a Mitochondria Isolation Kit (ThermoFisher) following the manufacturer’s protocol. For human MM cells (3 x 10^7^ cells/condition), mitochondria were fractionated from cells using 700 µl of Mitochondria Isolation Buffer (250 mM sucrose, 10 mM HEPES, pH7.5, 10 mM KCl, 1 mM EDTA and 0.1 mM EGTA with protease and phosphatase inhibitors) followed by homogenization in a Dounce homogenizer as previously described (40). Nuclei were pelleted from the Mitochondrial Isolation lysates by centrifugation at 1,000 g for 10 minutes at 4°C. The cleared lysates were then centrifuged at 10,000 g for 25 minutes at 4°C to obtain the pellets of mitochondria. The supernatants were further centrifuged at 100,000 g for 2 hours to separate the pellets of microsomes (rich in ER) from cytosolic proteins (S100 fraction). One-fifth volume of 5X SDS sample buffer was added into each S100 fraction. The pellets of mitochondria and microsomes (rich in ER) were lysed and sonicated in 300 μl of 2X SDS sample buffer, respectively. Cytoplasmic and nuclear extracts were prepared as previously described (15, 33). All protein samples were subsequently boiled for 10 minutes for immunoblot analyses.

### Co-Immunoprecipitation Assay of Mitochondrial Lysates

Human MM cell line 8226 cells (1.5 x 10^8^ cells/condition) transduced with pUB-TRAF3-SBP-6xHis or pUB-FLAG-TRAF3 were used for mitochondrial fractionation as described above. Mitochondrial pellets were lysed and sonicated in CHAPS lysis buffer (1% CHAPS, 20 mM Tris, pH 7.4, 150 mM NaCl, 50 mM β-glycerophosphate, and 5% glycerol with freshly added 1 mM DTT and EDTA-free Mini-complete protease inhibitor cocktail) (39). Mitochondrial lysates were cleared by centrifugation at 10,000 g for 20 minutes at 4°C. Cleared mitochondrial lysates were subsequently incubated with the Streptavidin-Sepharose beads (Pierce) to immunoprecipitate TRAF3-SBP-6xHis. Immunoprecipitates were washed 5 times with the Wash Buffer (39), resuspended in 2X SDS sample buffer, boiled for 10 minutes, and then separated on 4-16% gradient SDS-PAGE (Invitrogen) for mass spectrometry-based sequencing or immunoblot analyses.

### Liquid Chromatography-Tandem Mass Spectrometry (LC-MS/MS)-Based Sequencing

Mitochondrial lysates immunoprecipitated with the Streptavidin-Sepharose beads were used for LC-MS/MS-based sequencing. The entire gel lanes of TRAF3-SBP-6xHis and the negative control (FLAG-TRAF3) immunoprecipitates were each sectioned into 15 continuous slices. The gel slice samples were subjected to thiol reduction by TCEP, alkylation with iodoacetamide, and digestion with sequencing-grade modified trypsin (41, 42). Peptides were eluted from the gel slices, desalted, and then subjected to reversed-phase nano-flow ultra high performance capillary liquid chromatography (uPLC) followed by high-resolution/high-mass accuracy MS/MS analysis using an LC-MS platform consisting of an Eksigent Nano Ultra 2D Plus uPLC system hyphenated to a Thermo Orbi Velos mass spectrometer (40). The MS/MS was set to operate in data dependent acquisition mode using a duty cycle in which the top 15 most abundant peptide ions in the full scan MS were targeted for MS/MS sequencing. Full scan MS1 spectra were acquired at 100,000 resolving power and maintained mass calibration to within 2-3 ppm mass accuracy. LC-MS/MS data were searched against the human IPI and UniProt databases using the Mascot and Proteome Discoverer search engines (41, 42). Protein assignments were considered highly confident using a stringent false discovery rate threshold of <1%, as estimated by reversed database searching. Rough relative protein amounts were estimated using spectra counting values.

### Co-Immunoprecipitation From Transfected 293T Cells

For verification of the TRAF3-MFF interaction, 293T cells were co-transfected with pUB-TRAF3-SBP-6xHis and pcDNA3-Myc-MFF, pcDNA3-Myc-MFF3, pcDNA3-Myc-CSNK2A2 or an empty expression vector pcDNA3-Myc. At day 2 post transfection, cells (2 x 10^7^ cells/condition) were harvested and total cellular proteins were lysed in 1% CHAPS Lysis Buffer (39), sonicated and cleared by centrifugation. The cleared lysates were immunoprecipitated with the Streptavidin-Sepharose beads. Immunoprecipitates were washed 5 times with the Wash Buffer (39), resuspended in 2X SDS sample buffer, boiled for 10 minutes, and then separated on SDS-PAGE for immunoblot analyses.

### *In Vitro* Transcription and Translation of Myc-MFF

In vitro translation of Myc-MFF proteins was performed using the pcDNA3-Myc-MFF plasmid as the template with a coupled in vitro transcription/translation system from reticulocyte lysates (TnT^®^ Quick T7 Coupled Transcription/Translation System, Promega), following the manufacturer’s protocol. The reactions were incubated at 30°C for 90 min. Translation of Myc-MFF was verified by Western blot analysis.

### GST Pull-Down Assay

For preparation of GST-TRAF3 fusion proteins, each pGEX-GST-TRAF3 plasmid, including wild type (WT) and the deletion mutants, was transformed into *E. coli* BL21 bacteria. Expression of GST-TRAF3 fusion proteins were induced with 0.2 mM isopropyl-β-D-thiogalactoside (IPTG) for 2 hours as described (35). Bacterial pellets were lysed and sonicated in the Bacteria Lysis Buffer (20 mM Tris-HCl, pH 8.0 and 150 mM NaCl with freshly added 1 mg/ml lysozyme, 1% sarkosyl, 1 mM DTT and 1x EDTA-free Protease Inhibitor cocktail). Bacterial lysates were centrifuged at 10,000 g for 30 minutes to remove insoluble materials. Cleared bacterial lysates were diluted with 3 volumes of the Dilution Buffer (2% Triton X-100, 20 mM Tris, pH 8.0, 150 mM NaCl, 1 mM DTT and 1x EDTA-free Protease Inhibitor cocktail). GST-TRAF3 fusion proteins were subsequently purified from the diluted bacterial lysates using Glutathione–Sepharose 4B beads (GE Healthcare) according to the manufacturer’s protocol. After incubation with the lysates, the beads were washed five times in PBS containing 0.5% Triton X-100, and then eluted with 50 mM Tris-HCl (pH 8.0) containing 10 mM of reduced glutathione. The concentrations of eluted GST-TRAF3 proteins were determined by Bradford analysis, and then verified by SDS-PAGE and GelCode Blue staining by comparing to protein standards of known concentrations loaded on the same gel.

For GST pull-down assay of Myc-MFF proteins expressed in 293T cells, whole cell lysates were prepared from 293T cells (2 x 10^7^ cells/condition) transfected with pcDNA3-Myc-MFF or pcDNA3-Myc-MFF3 using the 1% CHAPS Lysis Buffer (39). For GST pull-down assay of *in vitro* translated Myc-MFF proteins, the translated proteins (4 x 50 µl of reactions/condition) were also lysed in the 1% CHAPS Lysis Buffer (39). 293T cell lysates or the *in vitro* translated proteins were cleared by centrifugation, and then incubated with Glutathione-Sepharose 4B beads for 1 hour at 4°C to remove non-specific bead interactors. The pre-cleared lysates were subsequently incubated with 10 µg of GST, GST-TRAF3 fusion protein or GST-TRAF3 deletion mutants in the presence of Glutathione-Sepharose 4B beads for 4 hours at 4°C. After incubation, the beads were washed 5 times with the Wash Buffer (39). Proteins pulled-down by the beads were eluted with 100 µl of 2X SDS sample buffer, boiled at 98°C for 10 minutes, and then analyzed by SDS-PAGE and immunoblot analyses. GST, GST-TRAF3 fusion protein or GST-TRAF3 deletion mutants in each pull-down sample were also analyzed by SDS-PAGE and visualized by GelCode blue staining. Band intensity of GelCode Blue stained gels was quantified using the ImageJ software (NIH, Bethesda, MD) (43).

### Electron Microscopy

For the electron microscopic (EM) examination of mitochondrial morphology and number, cells were fixed in 0.1 M cacodylate buffer with 2.5% glutaraldehyde, 4% paraformaldehyde and 8 mM CaCl_2_. The fixed and processed samples were subsequently analyzed on a JOEL 1200EX electron microscope as previously described (44).

### Mitochondrial Function Assay

Mitochondrial oxygen consumption rates (OCR) were measured using a Cell Mito Stress Test Kit and a Seahorse XFe24 Analyzer (Seahorse Bioscience, North Billerica, MA) as described previously (45). Briefly, purified splenic B cells were seeded at 2 x 10^6^ cells/well in XFe24 plates in Seahorse XF medium (10% FBS, 1% Pen-Strep). To derive different parameters of mitochondrial respiration, OCRs were measured sequentially before and after injecting oligomycin (a complex V inhibitor), p-trifluoromethoxy carbonyl cyanide phenyl hydrazine (FCCP, a protonophore and mitochondrial uncoupler), and antimycin A (a complex III inhibitor) plus rotenone (a complex I inhibitor), respectively, from XFe24 reagent ports. The following inhibitor concentrations were used for the mitochondrial stress test: oligomycin, 1 µM; FCCP, 1 µM; rotenone/antimycin A, 1 µM. All OCR measurements were normalized to cell number (per million cells).

### Mitochondrial ROS Analysis

For mitochondrial superoxide analysis, mouse splenic B cells were washed with PBS and stained with 1 µM of MitoSOX Red (Molecular Probes) for 30 minutes at 37°C in a 5% CO_2_ incubator. Stained cells were washed twice with pre-warmed PBS and subsequently analyzed by flow cytometry on a Northern Lights spectral flow cytometer.

### Ubiquitination Analysis

For ubiquitination analysis, 293T cells were co-transfected with LPC-HA-Ub and pUB-TRAF3-SBP-6xHis, pUB-Myc-MFF or an empty lentiviral vector pUB-Thy1.1. At day 2 post transfection, cells were treated with 10 µM of the proteasome inhibitor MG-132 at 37°C for 4 h, and then harvested at 2 x 10^7^ cells/condition. Total cellular proteins were lysed in 1% CHAPS Lysis Buffer (39) containing 1x Phosphatase Inhibitors (Pierce) and 1 mM NEM. The insoluble pellets were removed by centrifugation at 10,000 g for 20 minutes at 4°C. The CHAPS lysates were subsequently immunoprecipitated with the Anti-c-Myc Tag (9E10) Affinity Gel (BioLegend) or Streptavidin-Sepharose beads (Pierce). Immunoprecipitates were washed 5 times with the Wash Buffer (39) containing 1x Phosphatase Inhibitors and 1 mM NEM. Ubiquitination of the immunoprecipitated MFF or TRAF3 was analyzed by immunoblot analyses.

### Statistics

Statistical analyses were performed using the Prism software (GraphPad, La Jolla, CA). For direct comparison of the levels of cell apoptosis and mitochondrial parameters between LMC and *Traf3* ^-/-^ B cells, statistical significance was determined with the unpaired *t* test for two-tailed data. For comparison of three or more groups of data such as the relative binding between GST-TRAF3 (WT or mutants) and MFF, a one-way analysis of variance (ANOVA) was used to determine the statistical significance. *P* values less than 0.05 are considered significant, *P* values less than 0.01 are considered very significant, and *P* values less than 0.001 are considered highly significant.

## Results

### TRAF3 Promotes Mitochondria-Dependent Apoptosis in Resting B Cells

**Figure 1:**
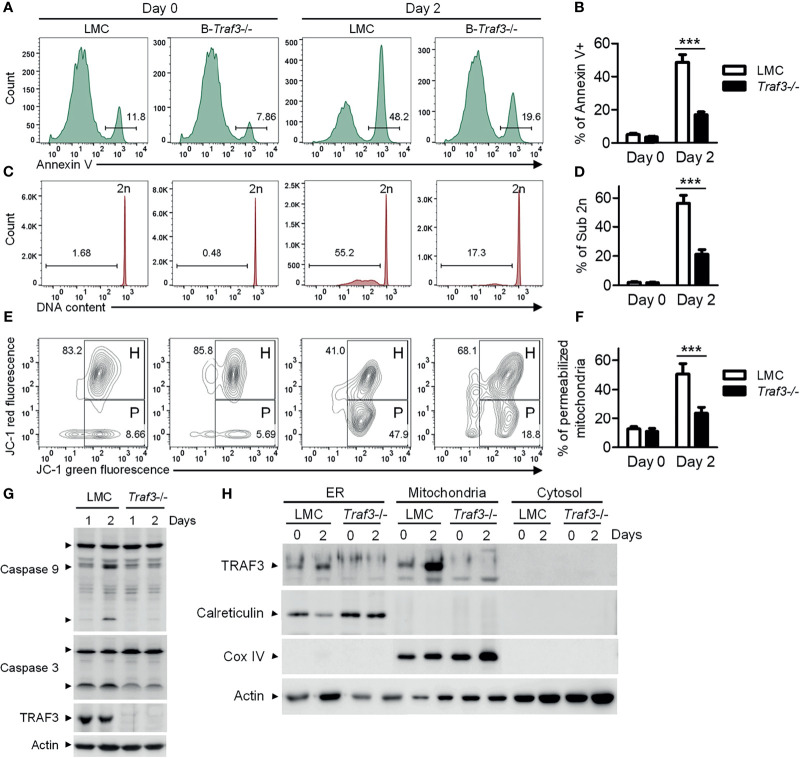
TRAF3 promoted the mitochondria-dependent intrinsic apoptotic pathway and was mainly localized at mitochondria in resting B cells. Splenic B cells were purified from gender-matched, young adult (8-12-week-old) naïve LMC or B-*Traf3* ^-/-^ mice. Purified cells were analyzed directly (Day 0) or at day 2 after *ex vivo* culture in mouse B cell medium. **(A)** Representative FACS profiles of annexin V staining. Gated populations indicate the annexin V+ apoptotic cells. **(B)** Graphical results of the percentage of annexin V+ apoptotic cells. **(C)** Representative FACS profiles of cell cycle distribution analyzed by PI staining. Gated populations indicate apoptotic cells with DNA fragmentation (DNA content < 2n). **(D)** Graphical results of the percentage of apoptotic cells with DNA fragmentation. **(E)** Representative FACS profiles of mitochondrial membrane permeabilization measured by MitoProbe JC-1 staining. Gated populations in H show cells with healthy mitochondria and gated populations in P indicate cells with permeabilized mitochondria. **(F)** Graphical results of the percentage of cells with permeabilized mitochondria. **(G)** Cleavage of caspase 9 and caspase 3. Total cellular proteins were prepared at day 1 or day 2 after culture and immunoblotted for caspase 9 and caspase 3, followed by TRAF3 and actin. **(H)** Cytoplasmic TRAF3 proteins were mainly localized at mitochondria in resting B cells. ER, mitochondrial and S100 (cytosolic) proteins were biochemically fractionated and analyzed by immunoblotting. Proteins in each fraction were immunoblotted for TRAF3, calreticulin (an ER protein), COX IV (a mitochondrial protein) and actin. Immunoblots shown are representative of 3 experiments. Graphs shown in **(B, D, F)** are the mean ± SD (n=6/group, including 3 female and 3 male samples). ***p < 0.001 by *t* test.

We previously reported that *Traf3* deficiency results in prolonged survival of mature B lymphocytes (15), which eventually leads to spontaneous development of splenic marginal zone lymphoma and B1 lymphoma in B-*Traf3* ^-/-^ mice (19). Interestingly, CD95-induced apoptosis is normal in *Traf3* ^-/-^ splenic B cells (15), suggesting that the extrinsic apoptotic pathway is not interrupted by *Traf3* deficiency. Here we measured the intrinsic apoptosis in premalignant *Traf3* ^-/-^ and TRAF3-sufficient splenic B cells prepared from tumor-free, young adult B-*Traf3* ^-/-^ and littermate control (LMC) mice. Approximately 50% of LMC B cells underwent apoptosis at day 2 after *ex vivo* culture as analyzed by annexin V staining (**Figures 1A, B**). DNA fragmentation was detected in the apoptotic LMC B cells by cell cycle analysis (**Figures 1C, D**). However, such cellular apoptosis and DNA fragmentation were drastically reduced in *Traf3* ^-/-^ B cells (**Figures 1A–D**). Mitochondria are the gateway of intrinsic apoptosis, which is often preceded by mitochondrial membrane potential change (30–32). We next analyzed the mitochondrial membrane potential changes using JC-1 staining and flow cytometry. We found that *Traf3* deficiency dramatically suppressed the mitochondrial membrane permeabilization in resting splenic B cells at day 2 after *ex vivo* culture (**Figures 1E, F**). Interestingly, treatment with the survival factor BAFF, which is known to induce TRAF3 degradation in B cells (28), was able to prevent apoptosis, DNA fragmentation and mitochondrial membrane permeabilization in LMC B cells but did not have detectable effects on *Traf3* ^-/-^ B cells (**Supplementary Figure 2**). We further investigated the downstream biochemical events induced by mitochondrial membrane permeabilization, including activation of the initiator caspase (caspase 9) and the effector caspase (caspase 3) of the intrinsic apoptotic pathway. We observed that activation of both caspase 9 and caspase 3, as demonstrated by the cleavage of both caspases, was substantially inhibited in *Traf3* ^-/-^ splenic B cells (**Figure 1G**). Taken together, these data indicate that after 2 days in culture and probably under other stress conditions, TRAF3 promotes the mitochondria-dependent intrinsic apoptotic pathway in resting splenic B cells.

It has been shown that *Traf3* deficiency leads to constitutive activation of NF-κB2 (15, 16), which controls the expression of the Bcl-2 family proteins (28, 46). Given that the Bcl-2 family proteins are important regulators of mitochondria-dependent apoptosis (47, 48), we examined the expression of several members of the Bcl-2 family in purified LMC and *Traf3* ^-/-^ splenic B cells. We detected a slight increase in the expression level of the anti-apoptotic protein Bcl-2 in *Traf3* ^-/-^ B cells at day 0 and a modest up-regulation in another anti-apoptotic protein Mcl-1 in *Traf3* ^-/-^ B cells at day 0 and day 1 after *ex vivo* culture in the absence of stimulation (**Supplementary Figure 3**). Interestingly, BAFF stimulation or CD40 ligation markedly induced up-regulation of the anti-apoptotic proteins Bcl-xL and Mcl-1 in both LMC and *Traf3* ^-/-^ B cells. Up-regulation of Bcl-xL and Mcl-1 induced by anti-CD40 was more robust than that induced by BAFF, while BAFF stimulation appeared to have a stronger effect on Bcl-xL and Mcl-1 up-regulation than *Traf3* deficiency in B cells (**Supplementary Figure 3**). However, *Traf3* deficiency was as potent as BAFF stimulation at inhibiting mitochondria-dependent apoptosis in resting splenic B cells (**Supplementary Figure 2**). These results suggest that in addition to the Bcl-2 family proteins, other mechanisms may also be involved in TRAF3-mediated regulation of mitochondria-dependent apoptosis in B cells.

### Cytoplasmic TRAF3 Is Mainly Localized at the Mitochondria in *Ex Vivo* Cultured Resting B Cells

In the absence of stimulation, cellular TRAF3 proteins are distributed in the cytoplasm and nucleus (28, 29) (**Supplementary Figure 4**). BAFF stimulation or CD40 ligation recruits TRAF3 from the cytoplasm to the BAFF receptor or CD40 signaling complex at the sphingolipid-enriched membrane rafts in B cells (49, 50). Viral infection leads to TRAF3 re-localization from the cytoplasm to mitochondria (51, 52). To determine if TRAF3 is evenly distributed within the cytoplasm in the absence of stimulation, we prepared ER, mitochondrial and cytosolic (S100) fractionations from resting splenic B cells of naïve mice. Unexpectedly, we found that the majority of cytoplasmic TRAF3 was in the mitochondrial fraction in resting splenic B cells (**Figure 1H**). We also noticed that in LMC B cells, TRAF3 proteins were remarkably up-regulated in the mitochondria and less robustly in the ER, but not up-regulated in the nucleus, at day 2 after *ex vivo* culture (**Figure 1H** and **Supplementary Figure 4**), probably because endogenous BAFF-induced TRAF3 degradation was eliminated after *ex vivo* culture. These data suggest that BAFF-induced recruitment and subsequent degradation of TRAF3 mainly affect the proteins localized at the mitochondria. Thus, the mitochondrial localization of TRAF3 raises an intriguing possibility that TRAF3 may also directly regulate mitochondrial physiology to induce mitochondria-dependent apoptosis in B cells.

### MFF Is a Novel TRAF3-Interacting Protein

Given that TRAF3 does not contain any mitochondrial targeting motif or transmembrane domain, we reasoned that TRAF3 may be associated with mitochondria in resting B cells by interacting with mitochondrial outer membrane (MOM) proteins. We sought to identify such TRAF3-binding partner(s) in the mitochondrial fraction of B cells using affinity purification followed by LC-MS/MS. To facilitate affinity purification, we generated lentiviral expression vectors of tagged TRAF3, including pUB-TRAF3-SBP-6xHis and pUB-FLAG-TRAF3 (39, 40). To strengthen the clinical relevance of our study and eliminate the interference of endogenous TRAF3 proteins, we used the human MM cell line 8226 that contains biallelic deletions of the *TRAF3* gene for transduction by the lentiviral expression vectors of tagged TRAF3. Transduction efficiency of each lentiviral vector in 8226 cells was > 90% as determined by FACS (**Supplementary Figure 5A**).

**Figure 2:**
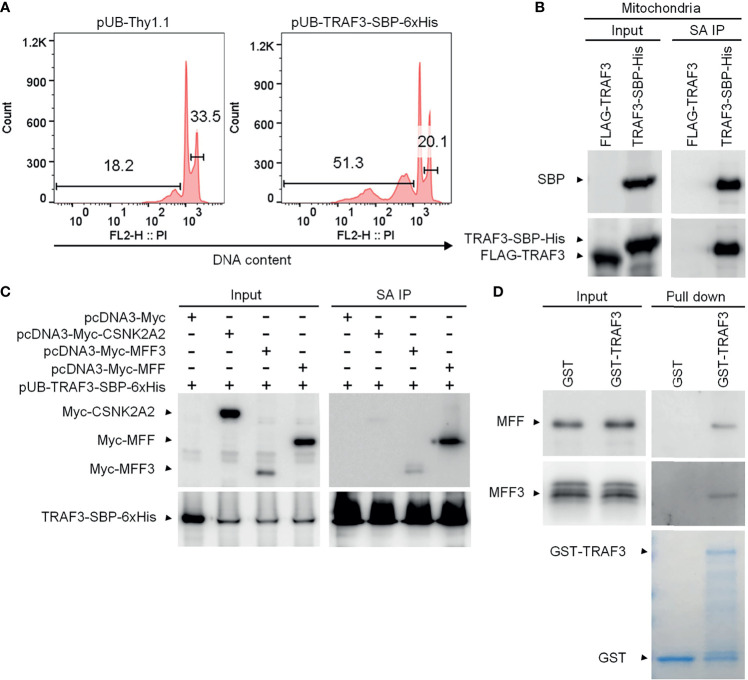
MFF is a novel TRAF3-interacting protein. **(A)** Validation of the functionality of C-terminally SBP-6xHis-tagged TRAF3 in human MM 8226 cells. Reconstitution with the lentiviral expression vector pUB-TRAF3-SBP-6xHis induced apoptosis in 8226 cells as demonstrated by the cell cycle analysis. Cells transduced with an empty lentiviral vector (pUB-Thy1.1) were used as a control. Cell cycle analysis was performed by PI staining and FACS at day 7 post transduction. Gated populations indicate apoptotic cells with DNA fragmentation (DNA content < 2n) and proliferating cells (2n < DNA content ≤ 4n). **(B)** Large scale affinity purification of SBP-6xHis-tagged TRAF3 from isolated mitochondria of transduced 8226 cells. Cells were harvested at day 4 post transduction and were fractionated to isolate mitochondria. Mitochondrial proteins were immunoprecipitated with Streptavidin (SA)-Sepharose beads. Immunoprecipitates of TRAF3-SBP-6xHis (SA IP) from the mitochondrial proteins were analyzed by immunoblotting and used to identify TRAF3-interacting proteins by LC-MS/MS-based sequencing. Immunoblots of mitochondrial proteins before immunoprecipitation were used as the input control. Cells transduced with FLAG-TRAF3 were used as a negative control for SA IP. **(C)** Co-immunoprecipitation of MFF and MFF3 with TRAF3. 293T cells were co-transfected with pUB-TRAF3-SBP-6xHis and pcDNA3-Myc-MFF, pcDNA3-Myc-MFF3, pcDNA3-Myc-CSNK2A2 or an empty expression vector pcDNA3-Myc. Transfected cells were harvested at day 2 post transfection and total cellular proteins were immunoprecipitated with Streptavidin-Sepharose beads. Immunoprecipitates (SA IP) were immunoblotted for the Myc tag followed by the SBP tag. Bands of Myc-tagged proteins are indicated. **(D)** MFF and MFF3 pulled down by GST-TRAF3. Pre-cleared whole cell lysates of 293T cells transfected with pcDNA3-Myc-MFF or pcDNA3-Myc-MFF3 were subjected to the pull-down assay by GST alone or GST-TRAF3 fusion protein in the presence of Glutathione-Sepharose beads. GST and GST-TRAF3 used for pull-down were analyzed by SDS-PAGE and visualized by GelCode blue staining (the bottom panel). Lysates that were purified by the same pull-down procedures with GST were used as a negative control. Myc-tagged MFF and MFF3 in the input lysates and pull-down proteins were detected by immunoblotting (the top panel). Results shown are representative of at least 3 independent experiments.

We first validated that the C-terminal streptavidin-binding peptide (SBP)-6xHis tag did not affect the function of TRAF3 as demonstrated by its potent induction of cellular apoptosis in transduced 8226 cells (**Figure 2A**), which was comparable to that induced by the untagged, native TRAF3 in transduced 8226 cells as we previously reported (25). We also verified that TRAF3-SBP-6xHis proteins maintained the mitochondrial localization of native TRAF3 (**Supplementary Figure 5B**). We next performed affinity purification using mitochondria isolated from transduced 8226 cells. We solubilized the mitochondrial proteins in 1% CHAPS lysis buffer (39, 40). Using Streptavidin-Sepharose beads, we immunoprecipitated TRAF3-SBP-6xHis and its associated proteins from the solubilized mitochondrial proteins. Cells transduced with FLAG-TRAF3 were subjected to the same biochemical fractionation and immunoprecipitation procedures as a negative control. We confirmed the high recovery rate of TRAF3-SBP-6xHis, but not FLAG-TRAF3, by immunoprecipitation with the Streptavidin-Sepharose beads using immunoblot analyses (**Figure 2B**). We subsequently performed LC-MS/MS of the mitochondrial proteins immunoprecipitated with the Streptavidin-Sepharose beads. Interestingly, we identified the MOM protein MFF (53) as a novel candidate TRAF3-interacting protein. However, our LC-MS/MS analysis did not detect any peptide derived from MAVS, a mitochondrial protein that is known to bind to TRAF3 upon viral infection (54, 55). In addition to MFF, our LC-MS/MS analysis identified a number of other candidate TRAF3-interacting proteins, including GSTP1, CKMT1A, PISD, SLC25A40, HDHD3, PYCARD, GPI, CDS2, DBI, ACAT2, RETSAT, PDE9A, SLC4A7, PRKG2, CDC42, ALDH3A1, ME1, CSNK2A2, CAP1, PGM2, PIK3R2, GSTO1, APEH, PSAT1, UGP2, GUCY1B3 and PRMT1. Bioinformatic analyses revealed that these proteins are predicted to be localized at the mitochondrial inner membrane (MIM), mitochondrial intermembrane space (MIS), mitochondrial matrix, ER membrane, plasma membrane or cytosol (UniProt: https://www.uniprot.org/). Lacking a mitochondrial targeting sequence or transmembrane domain, TRAF3 does not have direct access to the MIM/MIS/matrix under physiological conditions. We speculate that these MIM/MIS/matrix proteins identified in our LC-MS/MS analysis were likely pulled down with TRAF3 *via* post-lysis association. On the other hand, TRAF3-interacting proteins localized at the ER or plasma membrane or cytosol could not directly recruit TRAF3 to the mitochondria. We therefore selected to focus on the MOM protein MFF for further investigation.

There are multiple isoforms of human MFF generated by alternative splicing (53). MFF isoform 2 is a dominant isoform of MFF that is commonly expressed in various cell types, including epithelial cells, fibroblasts, prostate cancer cells and neuroblastoma cells as well as in the brain (56–61). Using PCR cloning, we found that two isoforms of MFF, MFF isoform 2 (291 aa; hereafter referred to as MFF) and MFF isoform 3 (MFF3; 243 aa), were expressed in human MM 8226 cells (**Supplementary Figure 6**). We therefore engineered expression vectors of Myc-tagged MFF and MFF3, and then performed co-immunoprecipitation (co-IP) experiments using 293T cells co-transfected with the expression vectors of TRAF3-SBP-6xHis and Myc-MFF or Myc-MFF3 to verify the interaction between TRAF3 and MFF. We also included Myc-tagged CSNK2A2, another protein identified by our affinity purification and LC-MS/MS, in the co-IP experiments for comparison. Our co-IP experiments verified the association between TRAF3 and MFF or MFF3 (**Figure 2C**). Interestingly, the interaction of TRAF3-MFF or TRAF3-MFF3 appeared to be much stronger than that observed in the TRAF3-CSNK2A2 co-IP (**Figure 2C**). These data demonstrate that MFF and MFF3 are associated with TRAF3 in transfected 293T cells.

### The TRAF-C Domain of TRAF3 Is Essential For Binding to MFF

We next investigated the potential direct binding between TRAF3 and MFF/MFF3 using two different GST pull-down assays. In the first assay, whole cell lysates of 293T cells transfected with Myc-tagged MFF or MFF3 were pre-cleared with Glutathione-Sepharose beads, and then incubated with purified GST-TRAF3 fusion proteins or GST native proteins in the presence of Glutathione-Sepharose beads. GST native proteins were used as a negative control to exclude proteins that were pulled down by GST but irrelevant to TRAF3. We did not detect non-specific binding between GST and MFF or MFF3 (**Figure 2D**). However, both MFF and MFF3 were pulled down by GST-TRAF3, indicating that there is a specific binding between TRAF3 and MFF or MFF3 (**Figure 2D**).

**Figure 3:**
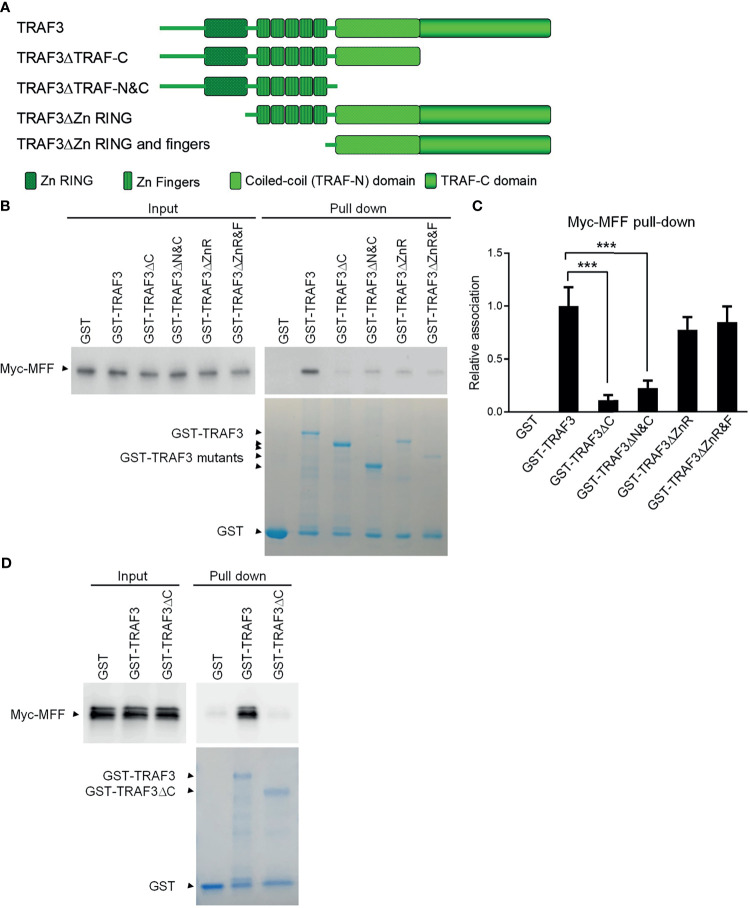
Mapping of the structural domains of TRAF3 required for the interaction with MFF. **(A)** Schematic diagram of the TRAF3 deletion mutants generated in this study and used for the mapping experiments. Structural domains of TRAF3 that were deleted are depicted in the figure. **(B)** Interaction with MFF determined by the GST pull-down assay. Pre-cleared whole cell lysates of 293T cells transfected with pcDNA3-Myc-MFF were used in the pull-down experiments by GST alone or GST fusion proteins of WT or deletion mutants of TRAF3 in the presence of Glutathione-Sepharose beads. GST, GST-TRAF3 and GST-TRAF3 deletion mutants used for pull-down were analyzed by SDS-PAGE and visualized by GelCode blue staining (the bottom panel). GST-TRAF3 deletion mutants examined include GST-TRAF3ΔTRAF-C (ΔC), GST-TRAF3ΔTRAF-N&C(ΔN&C), GST-TRAF3ΔZn RING (ΔZnR), and GST-TRAF3ΔZn RING and fingers (ΔZnR&F). Lysates that were purified by the same pull-down procedures with GST alone were used as a negative control and those with GST-TRAF3 were used as the positive control. Myc-tagged MFF in the input lysates and pull-down proteins were detected by immunoblotting (the top panel). Results shown are representative of 3 experiments. **(C)** Graphical results of the relative association between Myc-MFF and GST fusion proteins of WT or deletion mutants of TRAF3. The Myc-MFF bands on the immunoblots and GST-TRAF3 or mutant bands on the GelCode Blue stained gels in **(B)** were quantitated using a low-light imaging system and the ImageJ Software, respectively. The amount of Myc-MFF in each pull-down lane was first normalized to the intensity of the corresponding input Myc-MFF band and further normalized to the intensity of the corresponding GST-TRAF3 or mutant band. Fold of change of the normalized Myc-MFF pull-down relative to that detected for GST-TRAF3 WT was shown in the graph (mean ± SD, n = 3). ***p < 0.001 by one-way ANOVA. **(D)** Direct interaction between TRAF3 and MFF determined by GST pull-down assay of *in vitro* translated proteins. Pre-cleared protein lysates of *in vitro* translated Myc-MFF were used in the pull-down experiments by GST alone, GST-TRAF3 or GST-TRAF3ΔC in the presence of Glutathione-Sepharose beads. Myc-tagged MFF in the input lysates and pull-down proteins were detected by immunoblotting (the top panel). GelCode blue staining of GST, GST-TRAF3 and GST-TRAF3ΔC used for pull-down was shown in the bottom panel. Results shown are representative of 3 experiments.

Both MFF and MFF3 contain a coiled-coil domain (**Supplementary Figure 6**), which is known to mediate the interactions of other proteins with TRAFs, including the interactions of TRIP-TRAF2/1, p62-TRAF3 and T3JAM-TRAF3 (62–64). We did not find any other known TRAF-interacting motifs such as the (P/S/A/T)-X-(Q/E)-E or (P/S)XQX(T/S/D) motifs in the protein sequences of MFF or MFF3. To map the structural domains of TRAF3 required for its interaction with MFF, we performed GST pull-down experiments using GST fusion proteins of TRAF3 deletion mutants in comparison to wild type GST-TRAF3 with whole cell lysates of 293T cells transfected with Myc-tagged MFF as described above. Different GST-TRAF3 deletion mutants were examined, including GST-TRAF3ΔTRAF-C (with the TRAF-C domain deleted), GST-TRAF3ΔTRAF-N&C (with both the TRAF-N and TRAF-C domains deleted), GST-TRAF3ΔZnR (with the Zinc RING deleted) and GST-TRAF3ΔZnR&F (with the Zinc RING and all 5 Zinc Fingers deleted) (**Figure 3A**). GST, GST-TRAF3 and GST-TRAF3 deletion mutants used for the pull-down experiments were visualized by GelCode blue staining (**Figure 3B**). We found that the interaction between MFF and TRAF3 was severely impaired by the absence of the TRAF-C or TRAF-N&C domains (**Figures 3B, C**). In fact, the TRAF-C deletion almost completely abolished the interaction with MFF, while compound deletion of TRAF-N together with TRAF-C did not further compromise the interaction with MFF (**Figures 3B, C**). Thus, the TRAF-C domain appears to be essential for the binding between TRAF3 and MFF.

To further assess the potential direct binding between TRAF3 and MFF as well as the requirement of the TRAF-C domain in this interaction, we carried out the second GST pull-down assay using *in vitro* translated Myc-MFF proteins. Similar to the results described for the GST pull-down assay of Myc-MFF proteins expressed in 293T cells, we found that *in vitro* translated MFF proteins were specifically pulled down by GST-TRAF3 but not by GST-TRAF3ΔTRAF-C (**Figure 3D**). These data demonstrate that the TRAF-C domain is required for mediating the direct binding of TRAF3 to MFF. Taken together, we identified MFF as a novel TRAF3-interacting protein, prompting us to test the hypothesis that TRAF3 may directly regulate mitochondrial physiology impacted by MFF.

### TRAF3 Regulates the Morphology and Healthy Status of Mitochondria in B Cells

**Figure 4:**
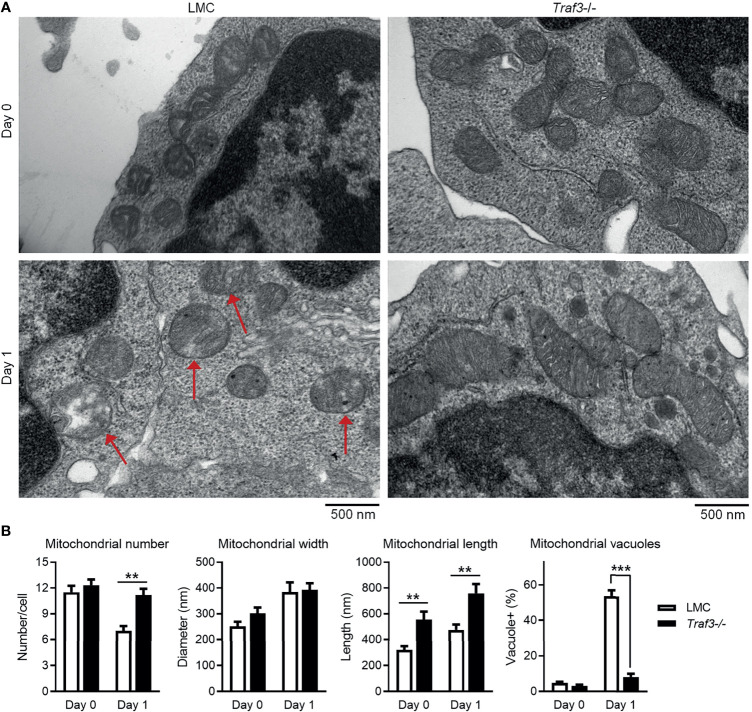
TRAF3 regulated the number and morphology of mitochondria in resting B cells. Splenic B cells were purified from gender-matched, young adult (8-12-week-old) naïve LMC or B-*Traf3* ^-/-^ mice. Purified cells were analyzed directly (Day 0) or at day 1 after *ex vivo* culture. The number and morphology of mitochondria in cells were analyzed by electron microscopic (EM) examination. **(A)** Representative EM micrographs of resting B cells. Sick mitochondria are indicated with red arrows in the figure. **(B)** Graphical results of the number, width and length of mitochondria as well as the percentage of cells containing sick mitochondria (vacuole+) determined by EM examination. Graphs shown are the mean ± SD (n = 6/group; **p < 0.01; ***p < 0.001 by *t* test).

The primary function of MFF is to promote mitochondrial fission, the division process of mitochondria within a cell that contributes to the regulation of mitochondrial number and morphology as well as quality (53, 65). Identification of MFF as a TRAF3-interacting protein led us to test the possibility that TRAF3 may regulate the number, morphology and quality of mitochondria in B cells. We thus compared the number and morphology of mitochondria between LMC and *Traf3* ^-/-^ resting splenic B cells using electron microscopy to discern potential changes caused by *Traf3* deficiency. We did not detect a significant difference in the number of mitochondria between the two genotypes of B cells freshly prepared from mouse spleens (**Figures 4A, B**). However, a significant decrease in mitochondrial number was observed in LMC but not in *Traf3* ^-/-^ B cells at day 1 after *ex vivo* culture (**Figures 4A, B**). Interestingly, we observed that premalignant *Traf3* ^-/-^ splenic B cells generally contained elongated mitochondria as demonstrated by their significantly increased mitochondrial length in comparison to LMC B cells (**Figures 4A, B**). Furthermore, after cultured *ex vivo* in the absence of survival factors for 1 day, the majority of mitochondria in LMC B cells exhibited abnormal and irregular vacuoles as well as loss of mitochondrial cristae, while *Traf3* ^-/-^ B cells maintained a healthy morphology of mitochondria (**Figures 4A, B**). These data thus indicate that under certain situations such as growth factor deprivation, TRAF3 regulates the number and morphology as well as healthy status of mitochondria in B lymphocytes.

### Regulation of Mitochondrial Respiration and ROS Production by TRAF3

**Figure 5:**
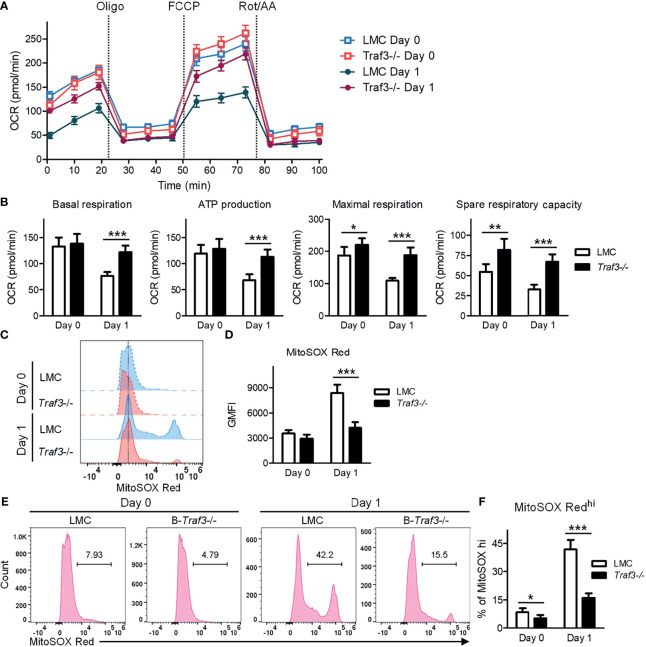
Regulation of mitochondrial function by TRAF3 in resting B cells. Splenic B cells were purified from gender-matched, young adult (8-12-week-old) naïve LMC or B-*Traf3* ^-/-^ mice. Purified cells were analyzed directly (Day 0) or at day 1 after *ex vivo* culture. **(A, B)** Mitochondrial respiration and energy production determined by the Seahorse Cell Mito Stress Test. **(A)** Kinetic changes of the oxygen consumption rate (OCR). Injection time points of oligomycin (Oligo), FCCP and rotenone/antimycin A (Rot/AA) are indicated in the figure. **(B)** Graphical results of the OCRs for basal respiration, ATP production, maximal respiration and spare respiratory capacity. **(C–F)** Mitochondrial superoxide levels analyzed MitoSOX Red staining and flow cytometry. **(C)** Representative FACS histogram overlay comparing the levels of MitoSOX Red staining intensity. **(D)** Graphical results of the geometric mean (GM) of MitoSOX Red fluorescence intensity (FI). **(E)** Representative FACS profiles showing the gated MitoSOX Red^hi^ populations. **(F)** Graphical results of the percentage of MitoSOX Red^hi^ subsets. **(B, D, F)** Graphs shown are the mean ± SD (n = 6/group; *p < 0.05; **p < 0.01; ***p < 0.001 by *t* test).

It has been shown that MFF-mediated mitochondrial fission not only regulates mitochondrial number and morphology, but also modulates important mitochondrial functions, including mitochondrial respiration and energy production (53, 65). We therefore examined these mitochondrial functions in LMC and *Traf3* ^-/-^ resting splenic B cells using the Seahorse Cell Mito Stress Test. Our results showed that mitochondrial basal respiration and ATP production were comparable between the two genotypes of resting B cells freshly prepared from mouse spleens (**Figures 5A, B**). However, at day 1 after *ex vivo* culture in the absence of B cell survival factors, LMC B cells exhibited significantly decreased mitochondrial basal respiration and ATP production as compared to *Traf3* ^-/-^ B cells (**Figures 5A, B**). Interestingly, we found that the mitochondrial maximal respiration and spare respiratory capacity were slightly elevated in *Traf3* ^-/-^ B cells as compared to LMC B cells freshly prepared from mouse spleens and that these differences were substantially further enlarged at day 1 after *ex vivo* culture (**Figures 5A, B**). Thus, TRAF3 inhibited mitochondrial respiration and energy production in *ex vivo* cultured B cells.

MFF-induced mitochondrial functional alterations may lead to increased production of reactive oxygen species (ROS) and consequently oxidative stress, causing deteriorating mitochondrial health (53, 65). To investigate if TRAF3 also regulates mitochondrial ROS production, we measured the levels of mitochondrial superoxide using MitoSOX Red staining followed by flow cytometry. Our FACS data revealed that the mitochondrial superoxide levels were slightly higher in LMC than in *Traf3* ^-/-^ B cells freshly prepared from mouse spleens as measured by the geometric mean (GM) of MitoSOX Red fluorescence intensity (FI) and the percentage of MitoSOX Red^hi^ cells (**Figures 5C–F**). The differences in the mitochondrial superoxide levels between the two genotypes of B cells were drastically enlarged at day 1 after *ex vivo* culture (**Figures 5C–F**). We also observed that BAFF treatment effectively inhibited mitochondrial ROS production in LMC B cells but not in *Traf3* ^-/-^ B cells (**Supplementary Figure 7**), consistent with the model that BAFF-induced TRAF3 recruitment and degradation would inhibit TRAF3-mediated regulation of mitochondrial ROS production. Taken together, our findings indicate that TRAF3 regulates mitochondrial respiration, energy production and ROS production in resting B cells after 2 days in culture.

### The TRAF3-MFF Interaction Affects the Modifications of MFF and TRAF3

**Figure 6:**
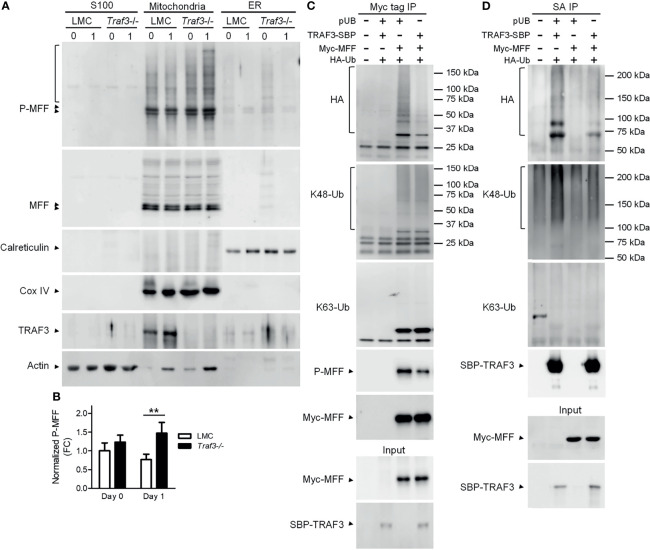
The TRAF3-MFF interaction affected the modifications of MFF and TRAF3. **(A, B)** Phosphorylation of MFF in resting B cells. Splenic B cells were purified from gender-matched, young adult (8-12-week-old) naïve LMC or B-*Traf3* ^-/-^ mice. Purified cells were analyzed directly (Day 0) or at day 1 after *ex vivo* culture. S100 (cytosolic), mitochondrial and ER proteins were biochemically fractionated and analyzed by immunoblotting. Proteins in each fraction were immunoblotted for phosphorylated MFF (P-MFF), total MFF, Calreticulin (an ER protein) and COX IV (a mitochondrial protein), followed by TRAF3 and actin. **(B)** Graphical results of the normalized P-MFF in the mitochondrial fraction. The phosphorylated and total MFF bands on the immunoblots in **(A)** were quantitated using a low-light imaging system. The intensity of P-MFF bands in each lane of the mitochondrial fraction was normalized to that of the corresponding total MFF bands. Fold of change of the normalized P-MFF relative to that detected for LMC B cells at Day 0 was shown in the graph (mean ± SD, n = 3). **p < 0.01 by *t* test. **(C, D)** Ubiquitination of MFF and TRAF3 was affected by the MFF-TRAF3 interaction in 293T cells. Cells were co-transfected with lentiviral expression vectors of HA-tagged ubiquitin (LPA-HA-Ub) and Myc-tagged MFF (pUB-Myc-MFF), SBP-tagged TRAF3 (pUB-TRAF3-SBP-6xHis) or an empty vector pUB-Thy1.1. Transfected cells were harvested at day 2 post transfection. Total cellular proteins were immunoprecipitated with Anti-c-Myc Tag (9E10) Affinity Gel **(C)** or Streptavidin-Sepharose beads **(D)**. Immunoprecipitates [Myc tag IP in **(C)** or SA IP in **(D)**] were immunoblotted for the HA tag, K48-Ub and K63-Ub to detect the ubiquitination, K48- or K63-linked polyubiquitination of MFF **(C)** and TRAF3 **(D)**, respectively. Phosphorylated MFF (P-MFF) was also analyzed in the Myc tag IP **(C)**. Immunoblotting of Myc-MFF and SBP-TRAF3 was performed with both the immunoprecipitates and input lysates (bottom panels). Immunoblots shown are representative of 3 experiments.

The activity of MFF is regulated by its post-translational modifications, including phosphorylation and ubiquitination (53, 65). In light of our evidence that TRAF3 regulates mitochondrial morphology and functions, we sought to analyze if the TRAF3-MFF interaction affects the modifications of MFF to modulate its activity. We first compared the phosphorylation of MFF in cytosolic (S100), mitochondrial and ER fractions between LMC and *Traf3* ^-/-^ resting splenic B cells. Consistent with the published literature, we observed that MFF proteins were predominantly localized at mitochondria in resting splenic B cells (**Figure 6A**). MFF phosphorylation was not significantly different between LMC and *Traf3* ^-/-^ B cells freshly prepared from mouse spleens (**Figures 6A, B**). However, MFF phosphorylation was significantly reduced in LMC B cells as compared to *Traf3* ^-/-^ B cells at day 1 after *ex vivo* culture in the absence of B cell survival factors (**Figures 6A, B**). These data suggest that up-regulation of mitochondrial TRAF3 protein levels may inhibit the phosphorylation of mitochondrial MFF in resting B cells.

We noticed that there were multiple bands of higher molecular weights in the immunoblots of phosphorylated MFF (P-MFF), which exhibited a regulation pattern similar to that described above for MFF phosphorylation and likely represent polyubiquitinated versions of P-MFF (**Figure 6A**). Given that TRAF3 is an E3 ubiquitin ligase (14, 28), we further investigated if the TRAF3-MFF interaction directly impacts the ubiquitination of MFF or TRAF3. We co-transfected lentiviral expression vectors of HA-tagged ubiquitin together with Myc-MFF and/or TRAF3-SBP-6xHis into 293T cells. At day 2 after transfection, ubiquitination of MFF and TRAF3 was analyzed by immunoprecipitation with Myc and SBP, respectively, followed by immunoblotting with anti-HA and ubiquitin (Ub)-specific Abs. We found that overexpression of TRAF3 inhibited both the ubiquitination and phosphorylation of MFF in transfected 293T cells as demonstrated by the HA and P-MFF immunoblots (**Figure 6C**), which is consistent with our results obtained from resting splenic B cells (**Figure 6A**). Subsequently, we attempted to interrogate the type of ubiquitin modification of MFF using Abs specific for K48- or K63-linked polyubiquitination. However, K48-linked polyubiquitination of MFF was not affected by TRAF3 overexpression, while K63-linked polyubiquitin chains were not detected in the Myc-MFF immunoprecipitates (**Figure 6C**), suggesting that TRAF3 inhibits MFF ubiquitination conjugated *via* a type distinct from the canonical K48- or K63-linked polyubiquitination. Interestingly, overexpression of MFF also inhibited the ubiquitination of TRAF3 in transfected 293T cells as demonstrated by the HA immunoblots (**Figure 6D**). Furthermore, we found that the decrease in TRAF3 ubiquitination was mainly attributable to the inhibition on K48-, but not K63-, linked polyubiquitination caused by MFF overexpression as revealed by the K48-Ub and K63-Ub immunoblots (**Figure 6D**). Taken together, our results showed that the TRAF3-MFF interaction inhibits the phosphorylation and ubiquitination of MFF as well as the K48-linked polyubiquitination of TRAF3. It has been shown that K48-linked polyubiquitination of TRAF3 induces the proteasome-dependent degradation of TRAF3 (28) and that phosphorylation and ubiquitination of MFF may increase its activities at promoting mitochondrial fission and facilitating mitophagy (56, 61, 66). In this context, our findings suggest that the TRAF3-MFF interaction may inhibit specific post-translational modifications of MFF and TRAF3 to modulate the activities of MFF and regulate the stability of TRAF3 in B cells.

### Overexpression of MFF Induces Mitochondria-Dependent Apoptosis in TRAF3-Deficient Human MM Cells

**Figure 7:**
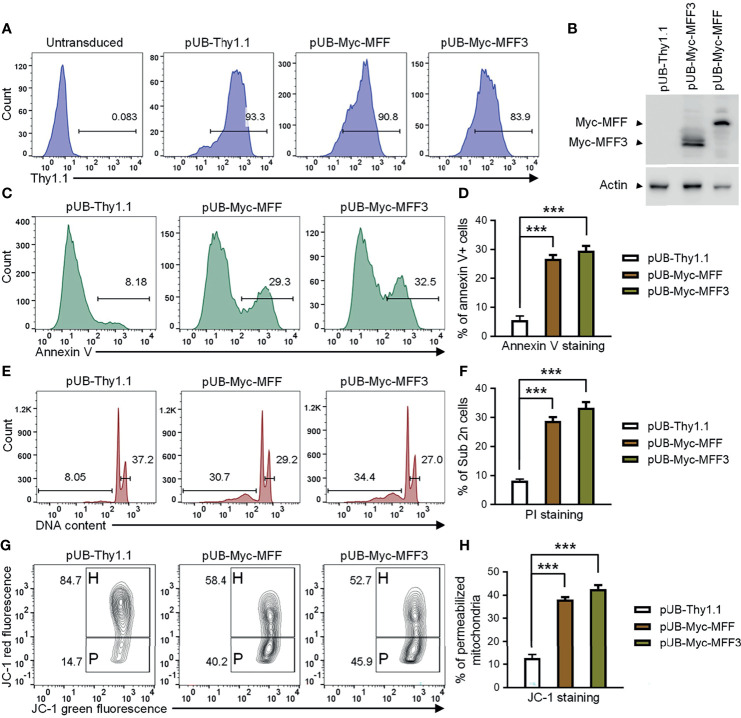
Overexpression of MFF induced mitochondria-dependent apoptosis in human MM 8226 cells. Cells were transduced with individual lentiviral expression vector of MFF (pUB-Myc-MFF), MFF3 (pUB-Myc-MFF3), or an empty vector (pUB-Thy1.1). **(A)** Transduction efficiency of 8226 cells analyzed by Thy1.1 immunofluorescence staining and FACS. Cells were analyzed at day 3 post transduction. Gated population (Thy1.1+) indicates the cells that were successfully transduced with the lentiviral expression vector. **(B)** Ectopic overexpression of MFF and MFF3 in transduced 8226 cells. Total cellular proteins were prepared at day 4 post transduction, and then immunoblotted for the Myc tag, followed by actin. Bands of Myc-MFF and Myc-MFF3 are indicated in the figure. **(C–H)** Cell apoptosis, DNA fragmentation and mitochondrial membrane permeabilization were analyzed at day 6 post transduction. **(C)** Representative FACS profiles of annexin V staining. Gated populations indicate the annexin V+ apoptotic cells. **(D)** Graphical results of the percentage of annexin V+ apoptotic cells. **(E)** Representative FACS profiles of cell cycle analysis by PI staining. Gated populations indicate apoptotic cells with DNA fragmentation (DNA content < 2n) and proliferating cells (2n < DNA content ≤ 4n). **(F)** Graphical results of the percentage of apoptotic cells with DNA fragmentation. **(G)** Representative FACS profiles of mitochondrial membrane permeabilization measured by MitoProbe JC-1 staining. Gated populations in H show cells with healthy mitochondria and gated populations in P indicate cells with permeabilized mitochondria. **(H)** Graphical results of the percentage of cells with permeabilized mitochondria. **(D, F, H)** The graphs depict the results of 3 independent experiments with duplicate samples in each experiment (mean ± SD). ***p < 0.001 by one-way ANOVA.

Our above evidence supports the hypothesis that TRAF3 may induce mitochondria-dependent apoptosis in B cells by regulating the activity of MFF. Altered activities of MFF or MFF overexpression have been shown to induce apoptosis in mammalian cells (53, 65). We reasoned that if MFF acts downstream of TRAF3, MFF overexpression may restore cellular apoptosis in TRAF3-deficient malignant B cells. To address this, we generated lentiviral expression vectors of MFF and MFF3, including pUB-Myc-MFF and pUB-Myc-MFF3. We used these lentiviral expression vectors to transduce human MM 8226 cells and ectopically overexpressed MFF or MFF3 proteins in the transduced cells. Cells transduced with an empty lentiviral expression vector (pUB-Thy1.1) were used as a control in these experiments. We determined the transduction efficiency as > 80% by FACS and verified the overexpression of Myc-tagged MFF and MFF3 proteins by immunoblot analyses (**Figures 7A, B**). We next investigated the functional consequences of ectopic overexpression of MFF and MFF3. We found that overexpression of MFF or MFF3 significantly induced cellular apoptosis in transduced 8226 cells as demonstrated by Annexin V staining (**Figures 7C, D**). Our cell cycle analysis revealed that MFF or MFF3 overexpression also induced DNA fragmentation in transduced 8226 cells (**Figures 7E, F**). To elucidate the mechanism of MFF-induced apoptosis, we further investigated whether it was mediated *via* the mitochondria-dependent pathway using JC-1 staining followed by flow cytometry. Our results showed that as compared to cells transduced with the empty lentiviral vector, overexpression of MFF or MFF3 markedly increased the percentage of 8226 cells containing permeabilized mitochondria (**Figures 7G, H**). These data are consistent with the hypothesis that MFF acts downstream of TRAF3 to promote intrinsic apoptosis. However, it is also possible that MFF overexpression may affect apoptosis *via* TRAF3-independent mechanisms such as causing an imbalance of the mitochondrial fission and fusion machineries in cells (53, 65, 67). Although the precise mechanisms remain to be elucidated, our results indicate that overexpression of MFF or MFF3 is able to induce mitochondria-dependent apoptosis in TRAF3-deficient malignant B cells, suggesting that MFF or mitochondrial fission machinery could be targetable points in human B cell malignancies, including those with *TRAF3* deletions or inactivating mutations.

### Genetic Alterations of *MFF* and *DNM1L* in Human B Cell Malignancies

MFF acts as a receptor of the fission protein dynamin-related protein 1 (Drp1; encoded by the *DNM1L* gene), a mechano-enzymatic GTPase, to recruit the cytosolic Drp1 onto the MOM surface, allowing Drp1 to assemble the mitochondrial constriction machinery at sites of ensuing fission (68–70). Drp1 subsequently hydrolyzes GTP to power membrane constriction and mitochondrial fission (69, 70). The functional importance of both MFF and Drp1 in mitochondrial physiology has been demonstrated by the evidence that genetic mutations of *MFF* and *DNM1L* play causal roles in patients with developmental abnormalities and neurological disorders (65, 71, 72). Given our current finding and the high frequency of deletions and mutations of *TRAF3* detected in human NHL and MM (13, 14), we searched the Cancer Genome Atlas (TCGA) (73) and the Catalog of Somatic Mutations in Cancer (COSMIC) (74) databases for the potential presence of genetic alterations of *MFF* and *DNM1L* in human B cell malignancies. We noticed that genetic alterations of *MFF* are very rare in human B cell cancers with only 0.49% (4 out of 819 cases) in pediatric acute lymphoid leukemia (ALL) identified as deep deletions (TARGET, 2018) (**Supplementary Figure 8A**). Compared to *MFF*, *DNM1L* exhibits relatively higher frequencies of genetic alterations in human B cell malignancies, including 7.1% (1/14) of mutations in NHL (75), 3.8% (2/53) of mutations in DLBCL (76), 0.63% (1/160) of mutations in CLL (77), 0.49% (1/205) of mutations in MM (78) and 0.24% (2/819) of amplifications in pediatric ALL (TARGET, 2018) (**Supplementary Figure 8B**). We next examined the combined genetic alterations of *MFF*, *DNM1L* and *TRAF3* in human B cell malignancies using the TCGA tool. Interestingly, we noticed that simultaneous genetic alterations of any two among these 3 genes are not documented in patients with B cell malignancies (**Supplementary Figure 9**). However, these genetic data are not conclusive due to the low incidence of mutations in *MFF* and *DNM1L* detected in human B cell malignancies and additional genetic alteration cases need to be analyzed to determine if TRAF3, MFF and Drp1 act in the same or overlapping pathways in B cells.

## Discussion

Mitochondria, the executioner organelle for cell death, are exploited by cancer cells and provide a validated therapeutic target in cancers (30–32). Mitochondria are dynamic, constantly undergoing fission and fusion to maintain their diverse functions (53, 65, 67). Mitochondrial dynamics is tightly controlled and crucial for the regulation of cell homeostasis and survival (53, 65, 67). Disruption or an imbalance of mitochondrial dynamics causes functional deterioration of mitochondria, often leading to cell apoptosis and a variety of human diseases ranging from neurodegenerative diseases to cancers (53, 65, 67). Therefore, proteins controlling mitochondrial fission have been recognized as essential regulators of mitochondrial functions, mitochondrial quality control, and cell apoptosis in health and diseases (53, 65, 67). In the present study, we identified the critical B cell survival regulator TRAF3 as a novel binding partner of the key mitochondrial fission protein, MFF, in B lymphocytes.

Elicited by our unexpected finding that the majority of cytoplasmic TRAF3 proteins were localized at the mitochondria in resting B cells after 2 days in culture, we identified MFF as a TRAF3-interacting protein using affinity purification of mitochondrial proteins isolated from human MM cells followed by LC-MS/MS-based sequencing. We verified the TRAF3-MFF interaction using co-immunoprecipitation and GST pull-down assays. In support of the model that the interaction with MFF allows TRAF3 to directly regulate the physiology of mitochondria, we obtained a variety of evidence in the present study. We demonstrated that in the absence of stimulation, increased protein levels of mitochondrial TRAF3 were associated with altered mitochondrial morphology, decreased mitochondrial respiration, increased mitochondrial ROS production and membrane permeabilization, which eventually culminated in caspase 9-dependent apoptotic pathway activation in resting B cells. In concordance with these findings, deletion of TRAF3 had the opposite effects on the morphology, function and healthy status of mitochondria as well as the mitochondria-dependent intrinsic apoptotic pathway in resting B cells. Interestingly, BAFF-induced degradation of TRAF3 appeared to mainly affect mitochondrial TRAF3, which would inhibit TRAF3-MFF-mediated regulation of mitochondrial function and mitochondria-dependent apoptosis in TRAF3-sufficient B cells. Indeed, BAFF stimulation inhibited mitochondrial ROS production and prevented mitochondria-dependent apoptosis in LMC but not in B-*Traf3* ^-/-^ splenic B cells. Taken together, our findings support the model that TRAF3 can directly regulate the physiology of mitochondria to promote the intrinsic apoptotic pathway *via* interacting with MFF.

Our findings help to understand some seemingly opposite roles of TRAF3 in B cell function and tumorigenesis. For example, not only *Traf3* deficiency leads to B lymphoma development in mice (19), transgenic overexpression of *TRAF3* in B cells also promotes B cell differentiation and mature non-Hodgkin lymphomas in mice (26, 27). Interestingly, the pro-tumorigenic activities of upregulated TRAF3 in B cells require simultaneous overexpression of the anti-apoptotic protein BCL-2 in B cells (27), suggesting a need for BCL-2-mediated protection of mitochondria in TRAF3-overexpressing B cells. In this context, it is conceivable that BCL-2 overexpression could counteract the TRAF3-MFF-mediated regulation of mitochondrial morphology and function, thereby preventing mitochondria-dependent apoptosis in TRAF3-overexpressing B cells.

Corroborating our evidence that ectopic overexpression of MFF induced mitochondria-dependent apoptosis in TRAF3-deficient malignant B cells, it has been previously reported that overexpression of MFF induced apoptosis and loss of MFF reduced apoptosis in other cell types such as epithelial cells, fibroblasts, cardiomyocytes, mesangial and HeLa cells (67–69, 79–83). Paradoxically, deficiency or silencing of *MFF* also induced apoptosis in cardiomyocytes, HeLa, prostate cancer and KRAS-transformed salivary duct cancer cells (58, 84, 85). These seemingly contradictory observations suggest that the protein and activity levels of MFF are tightly regulated in cells to achieve a delicate balance in mitochondrial dynamics and to appropriately control cell survival and apoptosis.

MFF recruits Drp1, the GTPase that executes mitochondrial fission, to the MOM to induce mitochondrial fission. In the present study, we could not detect co-immunoprecipitation of Drp1 with either MFF or TRAF3 under our experimental condition (data not shown). In addition, the protein levels of Drp1 in the S100, mitochondrial and ER fractions of resting splenic B cells were below the detection limit of immunoblot analyses in our experiments (data not shown). Therefore, we could not determine whether the TRAF3-MFF interaction facilitates or interferes with the recruitment of Drp1 by MFF. Detection of the weak interaction between Drp1 and MFF requires cross-linking prior to co-IP as reported in the literature (68, 86–89). Thus, the interaction between MFF and TRAF3 appears to be much stronger than that observed between MFF and Drp1, highlighting the importance of TRAF3 in modulating the functional properties of MFF. Indeed, we found that TRAF3 inhibits the phosphorylation and ubiquitination of MFF in resting B cells and co-transfected HEK 293T cells.

Phosphorylation of MFF by kinases such as AMPK, JNK, ERK1/2 or CK2α in different cellular contexts has been shown to increase the activity of MFF in recruiting Drp1 to mitochondria and promoting mitochondrial fission (56, 61, 66, 80, 90–94). Ubiquitination of MFF by the E3 ubiquitin ligase Parkin has been reported to promote the association between MFF and the autophagic adapter protein p62/SQSTM1, thereby facilitating mitophagy and clearance of damaged mitochondria (57). Alternatively, Parkin-mediated ubiquitination of MFF under non-stressed conditions regulates constitutive MFF turnover and induces the degradation of MFF in HEK 293T cells (95). Whether and how the TRAF3-MFF interaction affects the accessibility of MFF to its specific kinase (such as AMPK, JNK, ERK1/2 or CK2α) or E3 ubiquitin ligase (such as Parkin) in B cells await further investigation in future studies. Regardless of the detailed mechanisms, our results suggest that the TRAF3-MFF interaction has functional impacts on MFF modifications, mitochondrial morphology and function as well as mitochondria-dependent apoptosis in B cells.

However, it should be noted that our data do not exclude the possibility that TRAF3 may also indirectly regulate mitochondria-dependent apoptosis in B cells through additional MFF-independent mechanisms. In this regard, it has been shown that the NIK-NF-κB2 pathway is constitutively activated and nuclear CREB is markedly elevated in *Traf3* ^-/-^ splenic B cells (15, 16, 96), which lead to increased expression of anti-apoptotic proteins of the Bcl-2 family such as Bcl-2 and Mcl-1 (46, 96). The Bcl-2 family proteins are important regulators of mitochondrial physiology and intrinsic apoptotic pathways (47, 48). Moreover, we recently reported elevated Chkα-driven choline metabolism and increased levels of the phospholipids PC and PE in *Traf3* ^-/-^ splenic B cells (25, 97). PC and PE are the two most abundant phospholipids of mitochondrial membranes, critically regulating mitochondrial physiology and mitochondria-dependent apoptosis (98, 99). Therefore, it is likely that the TRAF3-MFF interaction together with the TRAF3-NIK-NF-κB2, TRAF3-CREB-Mcl-1 and TRAF3-Chkα-PC/PE pathways act cooperatively to regulate mitochondrial morphology, function and mitochondria-dependent apoptosis in normal B lymphocytes. Disruption of all these TRAF3-dependent mechanisms in TRAF3-deficient B cells leads to BAFF-independent survival and eventually contributes to B cell malignant transformation.

Interestingly, genetic alterations of the *TRAF3* gene are not limited to B cell malignancies but also detected in human epithelial cancers (14). Most notably, the human papilloma virus-positive (HPV+) head and neck squamous cell carcinomas (HNSCC) exhibit an exceptionally high frequency (~20%) of deep deletions and truncations of the *TRAF3* gene (14, 100, 101). Similar to that described for B cells, elevated activation of the NF-κB2 pathway is detected in TRAF3-deficient HNSCC cells (101). TRAF3 also plays pro-apoptotic roles in human bladder and colorectal carcinoma cells upon CD40 ligation *via* BAX/BAK-caspase 9- and ROS- dependent mechanisms (102, 103). So far, TRAF3-mediated pro-apoptotic effects in epithelial cells have been reported to be mediated *via* transcriptional regulation (101–103). In this study, we detected co-immunoprecipitation of MFF with TRAF3 in transfected 293T epithelial cells. In light of the similarity in the pro-apoptotic role of TRAF3 observed in B cells and epithelial cells, further investigation of the potential direct TRAF3-MFF interaction and its functional impacts in epithelial cancers represent an interesting area in future research. Such investigation would extend our knowledge on the potentially universal functions of the survival/apoptosis regulator TRAF3 in regulating mitochondria-dependent apoptosis.

In summary, our findings provide novel insights into the elaborate pathogenic mechanisms of TRAF3 inactivation-initiated B cell malignant transformation. Our study identified TRAF3 as a novel regulator of mitochondrial physiology in B lymphocytes and elucidated the TRAF3-MFF interaction as one of the underlying mechanisms. Our discovery of the TRAF3-MFF axis in B cells will open up new therapeutic opportunities for the treatment of human B cell malignancies, particularly those with *TRAF3* deletion or relevant mutations. We demonstrated that overexpression of MFF could restore the apoptotic pathway in TRAF3-deficient malignant B cells. Thus, an imbalance in mitochondrial dynamics, caused by either excessive or insufficient levels of MFF proteins or activities, could induce mitochondria-dependent apoptosis in malignant B cells. Interestingly, a cell permeable peptidomimetic MFF has been developed with demonstrated activity on killing melanoma, breast and lung cancer cells but not normal cells in preclinical models, indicating that MFF is an actionable therapeutic target in human cancers (59). Furthermore, pharmaceutical approaches that target mitochondrial dynamics have been developed for the treatment of neurodegenerative diseases and type 2 diabetes, including mitochondrial division inhibitor-1 (mdivi-1, a Drp1-specific inhibitor), dynasore, P110 and 15-oxospiramilactone, etc. (53, 65, 104). All these drugs can be exploited and repurposed to treat human B cell malignancies, overcome resistance to standard therapies and help to improve patient outcome, especially when given in combination with other available chemotherapies, radiotherapies and immunotherapies.

## Data Availability Statement

The original contributions presented in the study are included in the article/**Supplementary Material**. Further inquiries can be directed to the corresponding author.

## Ethics Statement

The animal study was reviewed and approved by Rutgers University.

## Author Contributions

PX, YL, and EW designed the experiments of this study. YL, SG, JJ, SZ, CL, DS, and PX performed the experiments and analyzed the data. SK carried out LC-MS/MS and analyzed the data. W-XZ supervised the ubiquitination studies. JG supervised the mitochondrial functional assays. HZ and EW provided guidance on the mitochondrial morphological examination. PX and YL wrote the initial manuscript. All authors contributed to the article and approved the submitted version.

## Funding

This study was supported by the Department of Defense grant W81XWH-13-1-0242 (PX), the National Institutes of Health grant R01 CA158402 (PX), a Pilot Award of Cancer Institute of New Jersey through Grant Number P30CA072720 from the National Cancer Institute (PX) and a Busch Biomedical Grant (PX). The FACS analyses were supported by the Flow Cytometry Core Facility with funding from NCI-CCSG P30CA072720.

## Conflict of Interest

The authors declare that the research was conducted in the absence of any commercial or financial relationships that could be construed as a potential conflict of interest.

## Publisher’s Note

All claims expressed in this article are solely those of the authors and do not necessarily represent those of their affiliated organizations, or those of the publisher, the editors and the reviewers. Any product that may be evaluated in this article, or claim that may be made by its manufacturer, is not guaranteed or endorsed by the publisher.

## References

[1] Morton LM Wang SS Devesa SS Hartge P Weisenburger DD Linet MS. Lymphoma Incidence Patterns by WHO Subtype in the United States, 1992-2001. Blood (2006) 107:265–76. doi: 10.1182/blood-2005-06-2508 PMC1895348 16150940

[2] Ruddon R. The Epidemiology of Human Cancer. In: Ruddon RW, editor. Cancer Biology, 4th ed. Oxford, England: Oxford University Press (2007). p. 62–116.

[3] Horner MJ Ries LAG Krapcho M Neyman N Aminou R Howlader N. Surveillance, Epidemiology, and End Results (SEER) Program. SEER Cancer Statistics Review, 1975-2006. Bethesda, MD: National Cancer Institute (2008). Available at: www.seer.cancer.gov. (Nov 2008 Sub).

[4] Miao Y Medeiros LJ Xu-Monette ZY Li J Young KH. Dysregulation of Cell Survival in Diffuse Large B Cell Lymphoma: Mechanisms and Therapeutic Targets. Front Oncol (2019) 9:107. doi: 10.3389/fonc.2019.00107 30881917 PMC6406015

[5] Adams CM Clark-Garvey S Porcu P Eischen CM. Targeting the Bcl-2 Family in B Cell Lymphoma. Front Oncol (2018) 8:636. doi: 10.3389/fonc.2018.00636 30671383 PMC6331425

[6] Pasqualucci L Zhang B. Genetic Drivers of NF-kappaB Deregulation in Diffuse Large B-Cell Lymphoma. Semin Cancer Biol (2016) 39:26–31. doi: 10.1016/j.semcancer.2016.08.001 27546290

[7] Waibel M Gregory G Shortt J Johnstone RW. Rational Combination Therapies Targeting Survival Signaling in Aggressive B-Cell Leukemia/Lymphoma. Curr Opin Hematol (2014) 21:297–308. doi: 10.1097/MOH.0000000000000045 24811162

[8] Voutsadakis IA. Apoptosis and the Pathogenesis of Lymphoma. Acta Oncol (2000) 39:151–6. doi: 10.1080/028418600430707 10859004

[9] Cillessen SA Meijer CJ Notoya M Ossenkoppele GJ Oudejans JJ. Molecular Targeted Therapies for Diffuse Large B-Cell Lymphoma Based on Apoptosis Profiles. J Pathol (2010) 220:509–20. doi: 10.1002/path.2670 20087881

[10] Muris JJ Meijer CJ Ossenkoppele GJ Vos W Oudejans JJ. Apoptosis Resistance and Response to Chemotherapy in Primary Nodal Diffuse Large B-Cell Lymphoma. Hematol Oncol (2006) 24:97–104. doi: 10.1002/hon.774 16715473

[11] Leslie LA Younes A. Targeting Oncogenic and Epigenetic Survival Pathways in Lymphoma. Leuk Lymphoma (2013) 54:2365–76. doi: 10.3109/10428194.2013.780288 23442067

[12] Spina V Rossi D. NF-kappaB Deregulation in Splenic Marginal Zone Lymphoma. Semin Cancer Biol (2016) 39:61–7. doi: 10.1016/j.semcancer.2016.08.002 27503810

[13] Moore CR Edwards SK Xie P. Targeting TRAF3 Downstream Signaling Pathways in B Cell Neoplasms. J Cancer Sci Ther (2015) 7:67–74. doi: 10.4172/1948-5956.1000327 25960828 PMC4422099

[14] Zhu S Jin J Gokhale S Lu A Shan H Feng J. Genetic Alterations of TRAF Proteins in Human Cancers. Front Immunol (2018) 9:2111. doi: 10.3389/fimmu.2018.02111 30294322 PMC6158389

[15] Xie P Stunz LL Larison KD Yang B Bishop GA. Tumor Necrosis Factor Receptor-Associated Factor 3 Is a Critical Regulator of B Cell Homeostasis in Secondary Lymphoid Organs. Immunity (2007) 27:253–67. doi: 10.1016/j.immuni.2007.07.012 PMC2084086 17723217

[16] Gardam S Sierro F Basten A Mackay F Brink R. TRAF2 and TRAF3 Signal Adapters Act Cooperatively to Control the Maturation and Survival Signals Delivered to B Cells by the BAFF Receptor. Immunity (2008) 28:391–401. doi: 10.1016/j.immuni.2008.01.009 18313334

[17] Keats JJ Fonseca R Chesi M Schop R Baker A Chng WJ. Promiscuous Mutations Activate the Noncanonical NF-kappaB Pathway in Multiple Myeloma. Cancer Cell (2007) 12:131–44. doi: 10.1016/j.ccr.2007.07.003 PMC2083698 17692805

[18] Annunziata CM Davis RE Demchenko Y Bellamy W Gabrea A Zhan F. Frequent Engagement of the Classical and Alternative NF-kappaB Pathways by Diverse Genetic Abnormalities in Multiple Myeloma. Cancer Cell (2007) 12:115–30. doi: 10.1016/j.ccr.2007.07.004 PMC2730509 17692804

[19] Moore CR Liu Y Shao CS Covey LR Morse HC 3rd Xie P. Specific Deletion of TRAF3 in B Lymphocytes Leads to B Lymphoma Development in Mice. Leukemia (2012) 26:1122–7. doi: 10.1038/leu.2011.309 PMC3433763 22033491

[20] Vince JE Wong WW Khan N Feltham R Chau D Ahmed AU. IAP Antagonists Target Ciap1 to Induce TNFalpha-Dependent Apoptosis. Cell (2007) 131:682–93. doi: 10.1016/j.cell.2007.10.037 18022363

[21] Varfolomeev E Blankenship JW Wayson SM Fedorova AV Kayagaki N Garg P. IAP Antagonists Induce Autoubiquitination of C-IAPs, NF-kappaB Activation, and TNFalpha-Dependent Apoptosis. Cell (2007) 131:669–81. doi: 10.1016/j.cell.2007.10.030 18022362

[22] Zarnegar BJ Wang Y Mahoney DJ Dempsey PW Cheung HH He J. Noncanonical NF-kappaB Activation Requires Coordinated Assembly of a Regulatory Complex of the Adaptors Ciap1, Ciap2, TRAF2 and TRAF3 and the Kinase NIK. Nat Immunol (2008) 9:1371–8. doi: 10.1038/ni.1676 PMC2676931 18997794

[23] Vallabhapurapu S Matsuzawa A Zhang W Tseng PH Keats JJ Wang H. Nonredundant and Complementary Functions of TRAF2 and TRAF3 in a Ubiquitination Cascade That Activates NIK-Dependent Alternative NF-kappaB Signaling. Nat Immunol (2008) 9:1364–70. doi: 10.1038/ni.1678 PMC2671996 18997792

[24] Gardam S Turner VM Anderton H Limaye S Basten A Koentgen F. Deletion of Ciap1 and Ciap2 in Murine B Lymphocytes Constitutively Activates Cell Survival Pathways and Inactivates the Germinal Center Response. Blood (2011) 117:4041–51. doi: 10.1182/blood-2010-10-312793 21300983

[25] Gokhale S Lu W Zhu S Liu Y Hart RP Rabinowitz JD. Elevated Choline Kinase Alpha-Mediated Choline Metabolism Supports the Prolonged Survival of TRAF3-Deficient B Lymphocytes. J Immunol (2020) 204:459–71. doi: 10.4049/jimmunol.1900658 PMC6946882 31826940

[26] Zapata JM Llobet D Krajewska M Lefebvre S Kress CL Reed JC. Lymphocyte-Specific TRAF3-Transgenic Mice Have Enhanced Humoral Responses and Develop Plasmacytosis, Autoimmunity, Inflammation, and Cancer. Blood (2009) 113:4595–603. doi: 10.1182/blood-2008-07-165456 PMC2680366 19074733

[27] Perez-Chacon G Adrados M Vallejo-Cremades MT Lefebvre S Reed JC Zapata JM. Dysregulated TRAF3 and BCL2 Expression Promotes Multiple Classes of Mature Non-Hodgkin B Cell Lymphoma in Mice. Front Immunol (2019) 9:3114. doi: 10.3389/fimmu.2018.03114 30687320 PMC6338067

[28] Xie P. TRAF Molecules in Cell Signaling and in Human Diseases. J Mol Signal (2013) 8:7. doi: 10.1186/1750-2187-8-7 23758787 PMC3697994

[29] Bishop GA Stunz LL Hostager BS. TRAF3 as a Multifaceted Regulator of B Lymphocyte Survival and Activation. Front Immunol (2018) 9:2161. doi: 10.3389/fimmu.2018.02161 30319624 PMC6165887

[30] Wang C Youle RJ. The Role of Mitochondria in Apoptosis*. Annu Rev Genet (2009) 43:95–118. doi: 10.1146/annurev-genet-102108-134850 19659442 PMC4762029

[31] Caroppi P Sinibaldi F Fiorucci L Santucci R. Apoptosis and Human Diseases: Mitochondrion Damage and Lethal Role of Released Cytochrome C as Proapoptotic Protein. Curr Med Chem (2009) 16:4058–65. doi: 10.2174/092986709789378206 19754424

[32] Burke PJ. Mitochondria, Bioenergetics and Apoptosis in Cancer. Trends Cancer (2017) 3:857–70. doi: 10.1016/j.trecan.2017.10.006 PMC5957506 29198441

[33] Edwards SK Moore CR Liu Y Grewal S Covey LR Xie P. N-Benzyladriamycin-14-Valerate (AD 198) Exhibits Potent Anti-Tumor Activity on TRAF3-Deficient Mouse B Lymphoma and Human Multiple Myeloma. BMC Cancer (2013) 13:481. doi: 10.1186/1471-2407-13-481 24131623 PMC3853153

[34] Li Y Franklin S Zhang MJ Vondriska TM. Highly Efficient Purification of Protein Complexes From Mammalian Cells Using a Novel Streptavidin-Binding Peptide and Hexahistidine Tandem Tag System: Application to Bruton’s Tyrosine Kinase. Protein Sci (2010) 20:140–9. doi: 10.1002/pro.546 PMC3047070 21080425

[35] Kiledjian M Day N Trifillis P. Purification and RNA Binding Properties of the Polycytidylate-Binding Proteins Alphacp1 and Alphacp2. Methods (1999) 17:84–91. doi: 10.1006/meth.1998.0710 10075886

[36] Zhou S Kurt-Jones EA Cerny AM Chan M Bronson RT Finberg RW. MyD88 Intrinsically Regulates CD4 T-Cell Responses. J Virol (2009) 83:1625–34. doi: 10.1128/JVI.01770-08 PMC2643758 19052080

[37] Pan JA Sun Y Jiang YP Bott AJ Jaber N Dou Z. TRIM21 Ubiquitylates SQSTM1/p62 and Suppresses Protein Sequestration to Regulate Redox Homeostasis. Mol Cell (2016) 61:720–33. doi: 10.1016/j.molcel.2016.02.007 PMC4779181 26942676

[38] Edwards SK Desai A Liu Y Moore CR Xie P. Expression and Function of a Novel Isoform of Sox5 in Malignant B Cells. Leuk Res (2014) 38:393–401. doi: 10.1016/j.leukres.2013.12.016 24418753 PMC3947564

[39] Edwards SK Han Y Liu Y Kreider BZ Grewal S Desai A. Signaling Mechanisms of Bortezomib in TRAF3-Deficient Mouse B Lymphoma and Human Multiple Myeloma Cells. Leuk Res (2016) 41:85–95. doi: 10.1016/j.leukres.2015.12.005 26740054 PMC4740239

[40] Edwards S Baron J Moore CR Liu Y Perlman DH Hart RP. Mutated in Colorectal Cancer (MCC) Is a Novel Oncogene in B Lymphocytes. J Hematol Oncol (2014) 7:56. doi: 10.1186/s13045-014-0056-6 25200342 PMC4172902

[41] Khan Z Amini S Bloom JS Ruse C Caudy AA Kruglyak L. Accurate Proteome-Wide Protein Quantification From High-Resolution 15N Mass Spectra. Genome Biol (2011) 12:R122. doi: 10.1186/gb-2011-12-12-r122 22182234 PMC3334617

[42] Ying W Perlman DH Li L Theberge R Costello CE McComb ME. Highly Efficient and Selective Enrichment of Peptide Subsets Combining Fluorous Chemistry With Reversed-Phase Chromatography. Rapid Commun Mass Spectrom (2009) 23:4019–30. doi: 10.1002/rcm.4343 PMC3584324 19924777

[43] Rueden CT Schindelin J Hiner MC DeZonia BE Walter AE Arena ET. ImageJ2: ImageJ for the Next Generation of Scientific Image Data. BMC Bioinformatics (2017) 18:529. doi: 10.1186/s12859-017-1934-z 29187165 PMC5708080

[44] Degenhardt K Mathew R Beaudoin B Bray K Anderson D Chen G. Autophagy Promotes Tumor Cell Survival and Restricts Necrosis, Inflammation, and Tumorigenesis. Cancer Cell (2006) 10:51–64. doi: 10.1016/j.ccr.2006.06.001 16843265 PMC2857533

[45] Guo JY Chen HY Mathew R Fan J Strohecker AM Karsli-Uzunbas G. Activated Ras Requires Autophagy to Maintain Oxidative Metabolism and Tumorigenesis. Genes Dev (2011) 25:460–70. doi: 10.1101/gad.2016311 PMC3049287 21317241

[46] Sen R. Control of B Lymphocyte Apoptosis by the Transcription Factor NF-kappaB. Immunity (2006) 25:871–83. doi: 10.1016/j.immuni.2006.12.003 17174931

[47] Leibowitz B Yu J. Mitochondrial Signaling in Cell Death via the Bcl-2 Family. Cancer Biol Ther (2010) 9:417–22. doi: 10.4161/cbt.9.6.11392 PMC2874116 20190564

[48] Martinou JC Youle RJ. Mitochondria in Apoptosis: Bcl-2 Family Members and Mitochondrial Dynamics. Dev Cell (2011) 21:92–101. doi: 10.1016/j.devcel.2011.06.017 21763611 PMC3156409

[49] Hostager BS Catlett IM Bishop GA. Recruitment of CD40 and Tumor Necrosis Factor Receptor-Associated Factors 2 and 3 to Membrane Microdomains During CD40 Signaling. J Biol Chem (2000) 275:15392–8. doi: 10.1074/jbc.M909520199 10748139

[50] Hildebrand JM Luo Z Manske MK Price-Troska T Ziesmer SC Lin W. A BAFF-R Mutation Associated With Non-Hodgkin Lymphoma Alters TRAF Recruitment and Reveals New Insights Into BAFF-R Signaling. J Exp Med (2010) 207:2569–79. doi: 10.1084/jem.20100857 PMC2989778 21041452

[51] Mao AP Li S Zhong B Li Y Yan J Li Q. Virus-Triggered Ubiquitination of TRAF3/6 by Ciap1/2 Is Essential for Induction of Interferon-Beta (IFN-Beta) and Cellular Antiviral Response. J Biol Chem (2010) 285:9470–6. doi: 10.1074/jbc.M109.071043 PMC2843197 20097753

[52] Huang Y Liu H Ge R Zhou Y Lou X Wang C. UXT-V1 Facilitates the Formation of MAVS Antiviral Signalosome on Mitochondria. J Immunol (2012) 188:358–66. doi: 10.4049/jimmunol.1102079 22131337

[53] Yu R Lendahl U Nister M Zhao J. Regulation of Mammalian Mitochondrial Dynamics: Opportunities and Challenges. Front Endocrinol (Lausanne) (2020) 11:374. doi: 10.3389/fendo.2020.00374 32595603 PMC7300174

[54] Saha SK Pietras EM He JQ Kang JR Liu SY Oganesyan G. Regulation of Antiviral Responses by a Direct and Specific Interaction Between TRAF3 and Cardif. EMBO J (2006) 25:3257–63. doi: 10.1038/sj.emboj.7601220 PMC1523175 16858409

[55] Paz S Vilasco M Werden SJ Arguello M Joseph-Pillai D Zhao T. A Functional C-Terminal TRAF3-Binding Site in MAVS Participates in Positive and Negative Regulation of the IFN Antiviral Response. Cell Res (2011) 21:895–910. doi: 10.1038/cr.2011.2 21200404 PMC3203699

[56] Toyama EQ Herzig S Courchet J Lewis TL Jr Loson OC Hellberg K. Metabolism. AMP-Activated Protein Kinase Mediates Mitochondrial Fission in Response to Energy Stress. Science (2016) 351:275–81. doi: 10.1126/science.aab4138 PMC4852862 26816379

[57] Gao J Qin S Jiang C. Parkin-Induced Ubiquitination of Mff Promotes Its Association With P62/SQSTM1 During Mitochondrial Depolarization. Acta Biochim Biophys Sin (Shanghai) (2015) 47:522–9. doi: 10.1093/abbs/gmv044 26008206

[58] Seo JH Agarwal E Chae YC Lee YG Garlick DS Storaci AM. Mitochondrial Fission Factor Is a Novel Myc-Dependent Regulator of Mitochondrial Permeability in Cancer. EBioMedicine (2019) 48:353–63. doi: 10.1016/j.ebiom.2019.09.017 PMC6838406 31542392

[59] Seo JH Chae YC Kossenkov AV Lee YG Tang HY Agarwal E. MFF Regulation of Mitochondrial Cell Death Is a Therapeutic Target in Cancer. Cancer Res (2019) 79:6215–26. doi: 10.1158/0008-5472.CAN-19-1982 PMC6911621 31582380

[60] Takamura H Koyama Y Matsuzaki S Yamada K Hattori T Miyata S. TRAP1 Controls Mitochondrial Fusion/Fission Balance Through Drp1 and Mff Expression. PloS One (2012) 7:e51912. doi: 10.1371/journal.pone.0051912 23284813 PMC3527369

[61] Ducommun S Deak M Sumpton D Ford RJ Nunez Galindo A Kussmann M. Motif Affinity and Mass Spectrometry Proteomic Approach for the Discovery of Cellular AMPK Targets: Identification of Mitochondrial Fission Factor as a New AMPK Substrate. Cell Signal (2015) 27:978–88. doi: 10.1016/j.cellsig.2015.02.008 25683918

[62] Lee SY Choi Y. TRAF-Interacting Protein (TRIP): A Novel Component of the Tumor Necrosis Factor Receptor (TNFR)- and CD30-TRAF Signaling Complexes That Inhibits TRAF2-Mediated NF-kappaB Activation. J Exp Med (1997) 185:1275–85. doi: 10.1084/jem.185.7.1275 PMC2196258 9104814

[63] Gamper C van Eyndhoven WG Schweiger E Mossbacher M Koo B Lederman S. TRAF-3 Interacts With P62 Nucleoporin, a Component of the Nuclear Pore Central Plug That Binds Classical NLS-Containing Import Complexes. Mol Immunol (2000) 37:73–84. doi: 10.1016/S0161-5890(00)00015-8 10781837

[64] Dadgostar H Doyle SE Shahangian A Garcia DE Cheng G. T3JAM, a Novel Protein That Specifically Interacts With TRAF3 and Promotes the Activation of JNK(1). FEBS Lett (2003) 553:403–7. doi: 10.1016/S0014-5793(03)01072-X 14572659

[65] El-Hattab AW Suleiman J Almannai M Scaglia F. Mitochondrial Dynamics: Biological Roles, Molecular Machinery, and Related Diseases. Mol Genet Metab (2018) 125:315–21. doi: 10.1016/j.ymgme.2018.10.003 30361041

[66] Seabright AP Fine NHF Barlow JP Lord SO Musa I Gray A. AMPK Activation Induces Mitophagy and Promotes Mitochondrial Fission While Activating TBK1 in a PINK1-Parkin Independent Manner. FASEB J (2020) 34:6284–301. doi: 10.1096/fj.201903051R PMC7212019 32201986

[67] Serasinghe MN Chipuk JE. Mitochondrial Fission in Human Diseases. Handb Exp Pharmacol (2017) 240:159–88. doi: 10.1007/164_2016_38 PMC6388405 28040850

[68] Otera H Wang C Cleland MM Setoguchi K Yokota S Youle RJ. Mff Is an Essential Factor for Mitochondrial Recruitment of Drp1 During Mitochondrial Fission in Mammalian Cells. J Cell Biol (2010) 191:1141–58. doi: 10.1083/jcb.201007152 PMC3002033 21149567

[69] Kornfeld OS Qvit N Haileselassie B Shamloo M Bernardi P Mochly-Rosen D. Interaction of Mitochondrial Fission Factor With Dynamin Related Protein 1 Governs Physiological Mitochondrial Function In Vivo. Sci Rep (2018) 8:14034. doi: 10.1038/s41598-018-32228-1 30232469 PMC6145916

[70] Singh S Sharma S. Dynamin-Related Protein-1 as Potential Therapeutic Target in Various Diseases. Inflammopharmacology (2017) 25:383–92. doi: 10.1007/s10787-017-0347-y 28409390

[71] Koch J Feichtinger RG Freisinger P Pies M Schrodl F Iuso A. Disturbed Mitochondrial and Peroxisomal Dynamics Due to Loss of MFF Causes Leigh-Like Encephalopathy, Optic Atrophy and Peripheral Neuropathy. J Med Genet (2016) 53:270–8. doi: 10.1136/jmedgenet-2015-103500 26783368

[72] Nasca A Nardecchia F Commone A Semeraro M Legati A Garavaglia B. Clinical and Biochemical Features in a Patient With Mitochondrial Fission Factor Gene Alteration. Front Genet (2018) 9:625. doi: 10.3389/fgene.2018.00625 30581454 PMC6292958

[73] Weinstein JN Collisson EA Mills GB Shaw KR Ozenberger BA Ellrott K. The Cancer Genome Atlas Pan-Cancer Analysis Project. Nat Genet (2013) 45:1113–20. doi: 10.1038/ng.2764 PMC3919969 24071849

[74] Forbes SA Bhamra G Bamford S Dawson E Kok C Clements J. The Catalogue of Somatic Mutations in Cancer (COSMIC). Curr Protoc Hum Genet (2008) Chapter 10:Unit 10 11. doi: 10.1002/0471142905.hg1011s57 PMC2705836 18428421

[75] Morin RD Mendez-Lago M Mungall AJ Goya R Mungall KL Corbett RD. Frequent Mutation of Histone-Modifying Genes in Non-Hodgkin Lymphoma. Nature (2011) 476:298–303. doi: 10.1038/nature10351 21796119 PMC3210554

[76] Morin RD Mungall K Pleasance E Mungall AJ Goya R Huff RD. Mutational and Structural Analysis of Diffuse Large B-Cell Lymphoma Using Whole-Genome Sequencing. Blood (2013) 122:1256–65. doi: 10.1182/blood-2013-02-483727 PMC3744992 23699601

[77] Landau DA Carter SL Stojanov P McKenna A Stevenson K Lawrence MS. Evolution and Impact of Subclonal Mutations in Chronic Lymphocytic Leukemia. Cell (2013) 152:714–26. doi: 10.1016/j.cell.2013.01.019 PMC3575604 23415222

[78] Lohr JG Stojanov P Carter SL Cruz-Gordillo P Lawrence MS Auclair D. Widespread Genetic Heterogeneity in Multiple Myeloma: Implications for Targeted Therapy. Cancer Cell (2014) 25:91–101. doi: 10.1016/j.ccr.2013.12.015 24434212 PMC4241387

[79] Gandre-Babbe S van der Bliek AM. The Novel Tail-Anchored Membrane Protein Mff Controls Mitochondrial and Peroxisomal Fission in Mammalian Cells. Mol Biol Cell (2008) 19:2402–12. doi: 10.1091/mbc.e07-12-1287 PMC2397315 18353969

[80] Sheng J Li H Dai Q Lu C Xu M Zhang J. DUSP1 Recuses Diabetic Nephropathy via Repressing JNK-Mff-Mitochondrial Fission Pathways. J Cell Physiol (2019) 234:3043–57. doi: 10.1002/jcp.27124 30191967

[81] Guido C Whitaker-Menezes D Lin Z Pestell RG Howell A Zimmers TA. Mitochondrial Fission Induces Glycolytic Reprogramming in Cancer-Associated Myofibroblasts, Driving Stromal Lactate Production, and Early Tumor Growth. Oncotarget (2012) 3:798–810. doi: 10.18632/oncotarget.574 22878233 PMC3478457

[82] Zhou H Hu S Jin Q Shi C Zhang Y Zhu P. Mff-Dependent Mitochondrial Fission Contributes to the Pathogenesis of Cardiac Microvasculature Ischemia/Reperfusion Injury via Induction of mROS-Mediated Cardiolipin Oxidation and HK2/VDAC1 Disassociation-Involved mPTP Opening. J Am Heart Assoc (2017) 6:1–20. doi: 10.1161/JAHA.116.005328 PMC5524036 28288978

[83] Ma Y Du M Yang F Mai Z Zhang C Qu W. Quantifying the Inhibitory Effect of Bcl-Xl on the Action of Mff Using Live-Cell Fluorescence Imaging. FEBS Open Bio (2019) 9:2041–51. doi: 10.1002/2211-5463.12739 PMC6886297 31587505

[84] Chen H Ren S Clish C Jain M Mootha V McCaffery JM. Titration of Mitochondrial Fusion Rescues Mff-Deficient Cardiomyopathy. J Cell Biol (2015) 211:795–805. doi: 10.1083/jcb.201507035 26598616 PMC4657172

[85] Lin HH Chung Y Cheng CT Ouyang C Fu Y Kuo CY. Autophagic Reliance Promotes Metabolic Reprogramming in Oncogenic KRAS-Driven Tumorigenesis. Autophagy (2018) 14:1481–98. doi: 10.1080/15548627.2018.1450708 PMC6135591 29956571

[86] Strack S Cribbs JT. Allosteric Modulation of Drp1 Mechanoenzyme Assembly and Mitochondrial Fission by the Variable Domain. J Biol Chem (2012) 287:10990–1001. doi: 10.1074/jbc.M112.342105 PMC3322891 22334657

[87] Frohlich C Grabiger S Schwefel D Faelber K Rosenbaum E Mears J. Structural Insights Into Oligomerization and Mitochondrial Remodelling of Dynamin 1-Like Protein. EMBO J (2013) 32:1280–92. doi: 10.1038/emboj.2013.74 PMC3642683 23584531

[88] Liu R Chan DC. The Mitochondrial Fission Receptor Mff Selectively Recruits Oligomerized Drp1. Mol Biol Cell (2015) 26:4466–77. doi: 10.1091/mbc.E15-08-0591 PMC4666140 26446846

[89] Clinton RW Francy CA Ramachandran R Qi X Mears JA. Dynamin-Related Protein 1 Oligomerization in Solution Impairs Functional Interactions With Membrane-Anchored Mitochondrial Fission Factor. J Biol Chem (2016) 291:478–92. doi: 10.1074/jbc.M115.680025 PMC4697186 26578514

[90] Long A Klimova N Kristian T. Mitochondrial NUDIX Hydrolases: A Metabolic Link Between NAD Catabolism, GTP and Mitochondrial Dynamics. Neurochem Int (2017) 109:193–201. doi: 10.1016/j.neuint.2017.03.009 28302504

[91] Zong Y Zhang CS Li M Wang W Wang Z Hawley SA. Hierarchical Activation of Compartmentalized Pools of AMPK Depends on Severity of Nutrient or Energy Stress. Cell Res (2019) 29:460–73. doi: 10.1038/s41422-019-0163-6 PMC6796943 30948787

[92] Jieensinue S Zhu H Li G Dong K Liang M Li Y. Tanshinone IIA Reduces SW837 Colorectal Cancer Cell Viability via the Promotion of Mitochondrial Fission by Activating JNK-Mff Signaling Pathways. BMC Cell Biol (2018) 19:21. doi: 10.1186/s12860-018-0174-z 30253740 PMC6157045

[93] Lu YT Li LZ Yang YL Yin X Liu Q Zhang L. Succinate Induces Aberrant Mitochondrial Fission in Cardiomyocytes Through GPR91 Signaling. Cell Death Dis (2018) 9:672. doi: 10.1038/s41419-018-0708-5 29867110 PMC5986788

[94] Zhou H Wang J Zhu P Zhu H Toan S Hu S. NR4A1 Aggravates the Cardiac Microvascular Ischemia Reperfusion Injury Through Suppressing FUNDC1-Mediated Mitophagy and Promoting Mff-Required Mitochondrial Fission by CK2alpha. Basic Res Cardiol (2018) 113:23. doi: 10.1007/s00395-018-0682-1 29744594

[95] Lee L Seager R Nakamura Y Wilkinson KA Henley JM. Parkin-Mediated Ubiquitination Contributes to the Constitutive Turnover of Mitochondrial Fission Factor (Mff). PloS One (2019) 14:e0213116. doi: 10.1371/journal.pone.0213116 31112535 PMC6528996

[96] Mambetsariev N Lin WW Stunz LL Hanson BM Hildebrand JM Bishop GA. Nuclear TRAF3 Is a Negative Regulator of CREB in B Cells. Proc Natl Acad Sci USA (2016) 113:1032–7. doi: 10.1073/pnas.1514586113 PMC4743771 26755589

[97] Gokhale S Xie P. ChoK-Full of Potential: Choline Kinase in B Cell and T Cell Malignancies. Pharmaceutics (2021) 13:1–15. doi: 10.3390/pharmaceutics13060911 PMC8234087 34202989

[98] Basu Ball W Neff JK Gohil VM. The Role of Nonbilayer Phospholipids in Mitochondrial Structure and Function. FEBS Lett (2018) 592:1273–90. doi: 10.1002/1873-3468.12887 PMC5918238 29067684

[99] van der Veen JN Kennelly JP Wan S Vance JE Vance DE Jacobs RL. The Critical Role of Phosphatidylcholine and Phosphatidylethanolamine Metabolism in Health and Disease. Biochim Biophys Acta Biomembr (2017) 1859:1558–72. doi: 10.1016/j.bbamem.2017.04.006 28411170

[100] Network TCGA. Comprehensive Genomic Characterization of Head and Neck Squamous Cell Carcinomas. Nature (2015) 517:576–82. doi: 10.1038/nature14129 PMC4311405 25631445

[101] Zhang J Chen T Yang X Cheng H Spath SS Clavijo PE. Attenuated TRAF3 Fosters Activation of Alternative NF-kappaB and Reduced Expression of Antiviral Interferon, TP53, and RB to Promote HPV-Positive Head and Neck Cancers. Cancer Res (2018) 78:4613–26. doi: 10.1158/0008-5472.CAN-17-0642 PMC7983169 29921694

[102] Georgopoulos NT Steele LP Thomson MJ Selby PJ Southgate J Trejdosiewicz LK. A Novel Mechanism of CD40-Induced Apoptosis of Carcinoma Cells Involving TRAF3 and JNK/AP-1 Activation. Cell Death Differ (2006) 13:1789–801. doi: 10.1038/sj.cdd.4401859 16429118

[103] Dunnill CJ Ibraheem K Mohamed A Southgate J Georgopoulos NT. A Redox State-Dictated Signalling Pathway Deciphers the Malignant Cell Specificity of CD40-Mediated Apoptosis. Oncogene (2017) 36:2515–28. doi: 10.1038/onc.2016.401 PMC5422712 27869172

[104] Rovira-Llopis S Banuls C Diaz-Morales N Hernandez-Mijares A Rocha M Victor VM. Mitochondrial Dynamics in Type 2 Diabetes: Pathophysiological Implications. Redox Biol (2017) 11:637–45. doi: 10.1016/j.redox.2017.01.013 PMC5284490 28131082

