# Supplementary material for: Mitochondrial Fission Factor Is a Novel Interacting Protein of the Critical B Cell Survival Regulator TRAF3 in B Lymphocytes
Source: Front Immunol. 2021 Oct 20;12:670338. doi: 10.3389/fimmu.2021.670338 (PMC8564014; doi:10.3389/fimmu.2021.670338)
Supplement: Supplementary file 1 [file DataSheet_1.pdf]

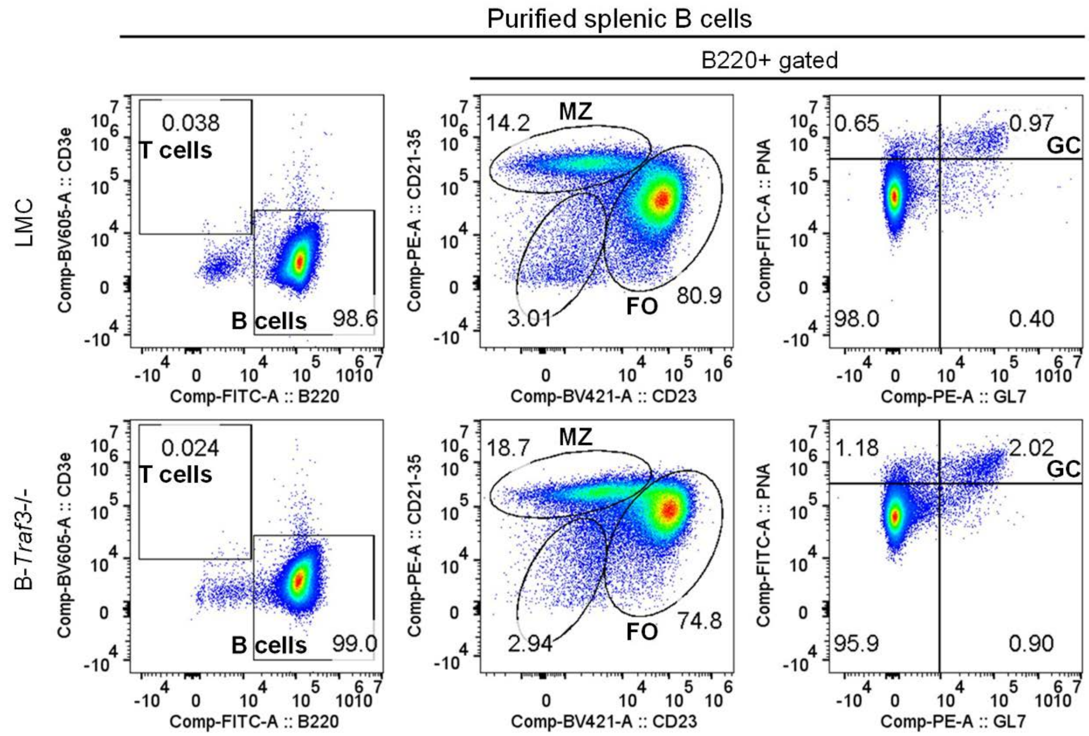

Supplementary Figure 1: FACS analysis of purified splenic B cells. Splenic B cells were purified from gender-matched, young adult (8-12-week-old) naïve LMC or B-*Traf3*$^{-/-}$ mice. Purified cells were analyzed directly (Day 0) by FACS analysis. Gated populations include B220+CD3- B cells, B220-CD3+ T cells, B220+CD21^{int}CD23+ follicular (FO) B cells, B220+CD21+CD23^{int} marginal zone (MZ) B cells, B220+GL7+PNA+ germinal center (GC) B cells. FACS profiles shown are representative of 3 experiments.

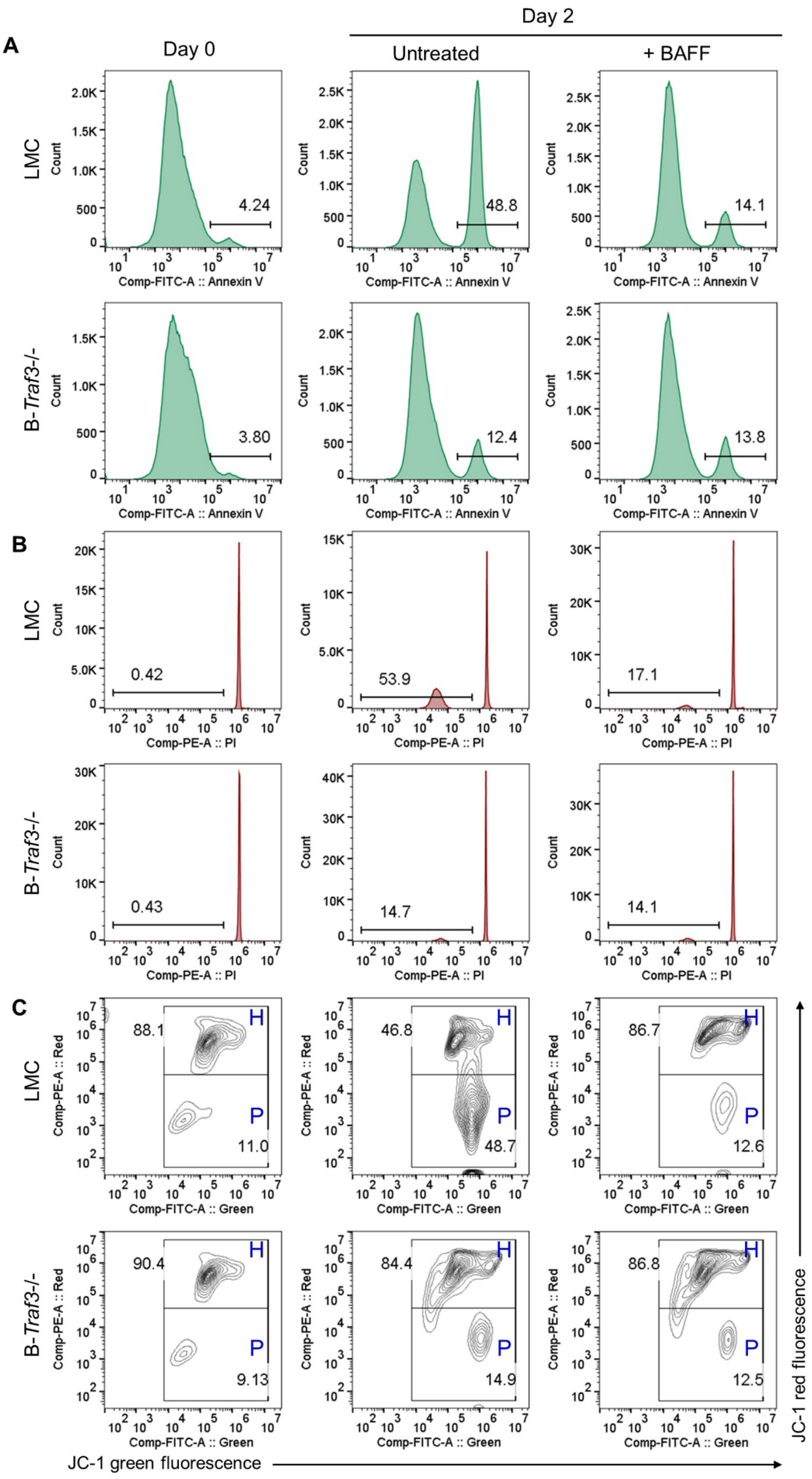

Supplementary Figure 2: BAFF treatment protected LMC B cells from apoptosis. Splenic B cells were purified from gender-matched, young adult (8-12-week-old) naïve LMC or B-*Traf3*$^{-/-}$ mice. Purified cells were analyzed directly (Day 0) or at day 2 after *ex vivo* culture in mouse B cell medium, in the absence or presence of 0.5 µg/ml BAFF. (**A**) Representative FACS profiles of annexin V staining. Gated populations indicate the annexin V+ apoptotic cells. (**B**) Representative FACS profiles of cell cycle distribution analyzed by PI staining. Gated populations indicate apoptotic cells with DNA fragmentation (DNA content < 2n). (**C**) Representative FACS profiles of mitochondrial membrane permeabilization measured by MitoProbe JC-1 staining. Gated populations in H show cells with healthy mitochondria and gated populations in P indicate cells with permeabilized mitochondria. FACS profiles shown are representative of 3 experiments.

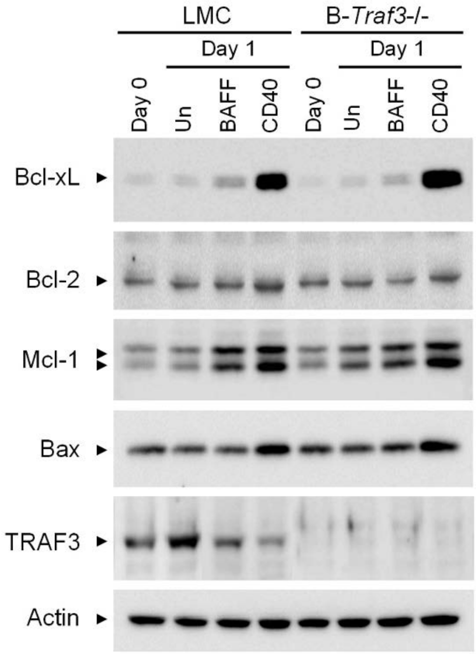

Supplementary Figure 3: Expression of the Bcl-2 family proteins was regulated by TRAF3, BAFF stimulation and CD40 ligation in splenic B cells. Splenic B cells were purified from gender-matched, young adult (8-12-week-old) naïve LMC or B-*Traf3*$^{-/-}$ mice. Purified cells were analyzed directly (Day 0) or at day 1 after *ex vivo* culture in mouse B cell medium, in the absence or presence of 0.5 µg/ml BAFF or 2 µg/ml of agonistic anti-CD40 (CD40). Total cellular proteins were immunoblotted for Bcl-xL, Bcl-2, Mcl-1 and Bax, followed by TRAF3 and actin. Immunoblots shown are representative of 3 experiments.

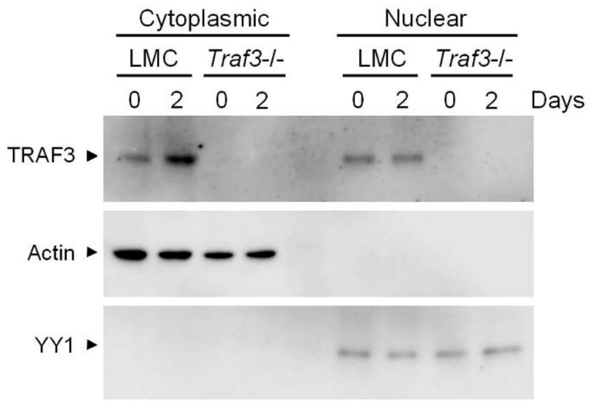

Supplementary Figure 4: Cellular TRAF3 proteins were distributed in the cytoplasm and nucleus in resting splenic B cells. Splenic B cells were purified from gender-matched, young adult (8-12-week-old) naïve LMC or B-*Traf3*$^{-/-}$ mice. Cytoplasmic and nuclear extracts were prepared at day 0 or day 2 after *ex vivo* culture in the absence of stimulation, and immunoblotted for TRAF3, followed by actin and YY1 (a nuclear protein). Immunoblots shown are representative of 3 experiments.

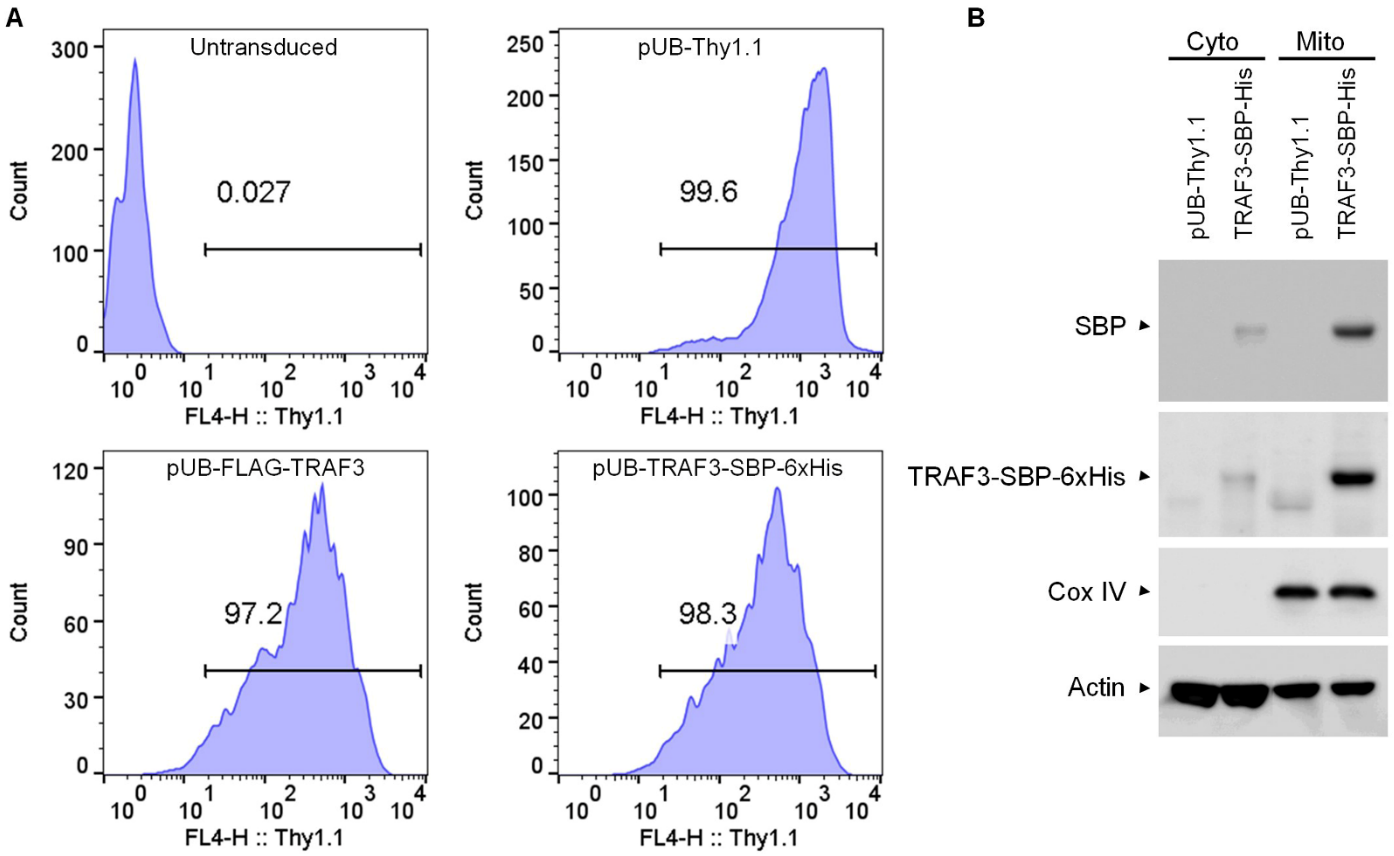

Supplementary Figure 5: Transduction efficiency and mitochondrial localization of SBP-6xHis-tagged TRAF3 in transduced human MM 8226 cells. Cells were transduced with individual lentiviral expression vector of SBP-6xHis-tagged TRAF3 (pUB-TRAF3-SBP-6xHis), FLAG-tagged TRAF3 (pUB-FLAG-TRAF3) or an empty vector (pUB-Thy1.1). (**A**) Representative FACS profiles of transduction efficiency. Transduction efficiency of 8226 cells was analyzed by Thy1.1 immunofluorescence staining and FACS at day 3 post transduction. Gated population (Thy1.1+) indicates the cells that were successfully transduced with the lentiviral expression vector. (**B**) TRAF3-SBP-6xHis proteins were mainly localized at mitochondria in transduced 8226 cells. Cytosolic (Cyto) and mitochondrial (Mito) proteins were biochemically fractionated and analyzed by immunoblotting. Proteins in each fraction were immunoblotted for the SBP tag, TRAF3, COX IV (a mitochondrial protein) and actin. FACS profiles and immunoblots shown are representative of 3 independent experiments.

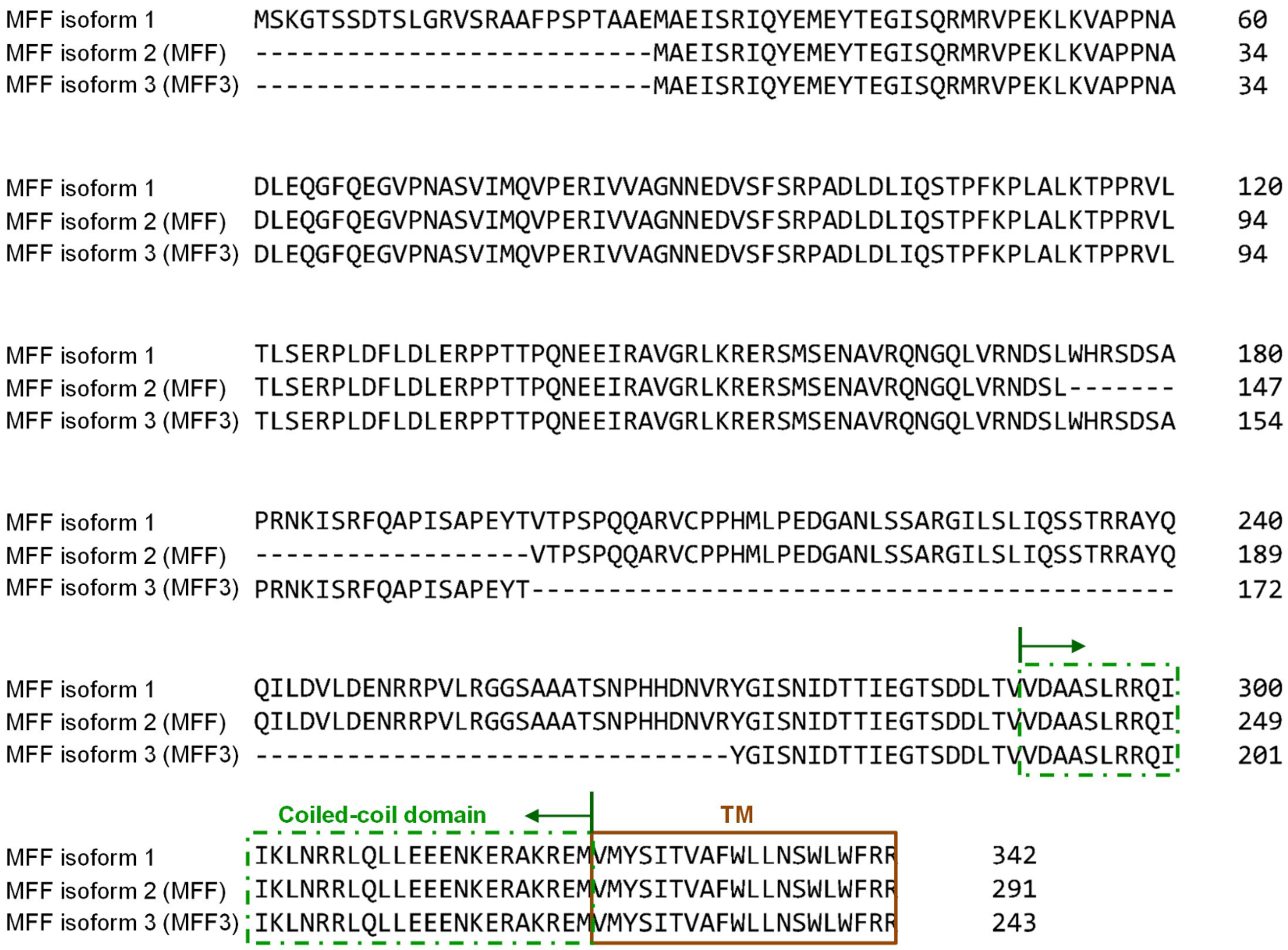

Supplementary Figure 6: The protein sequences of human MFF isoforms. The amino acid sequences of human MFF isoform 2 (MFF) and isoform 3 (MFF3) cloned from 8226 cells in the present study are aligned with that of MFF isoform 1 for comparison. The length of each protein is indicated in the figure. The coiled-coil domain of the three isoforms is highlighted with a dashed green box, while the transmembrane domain (TM) in shown in a solid brown box.

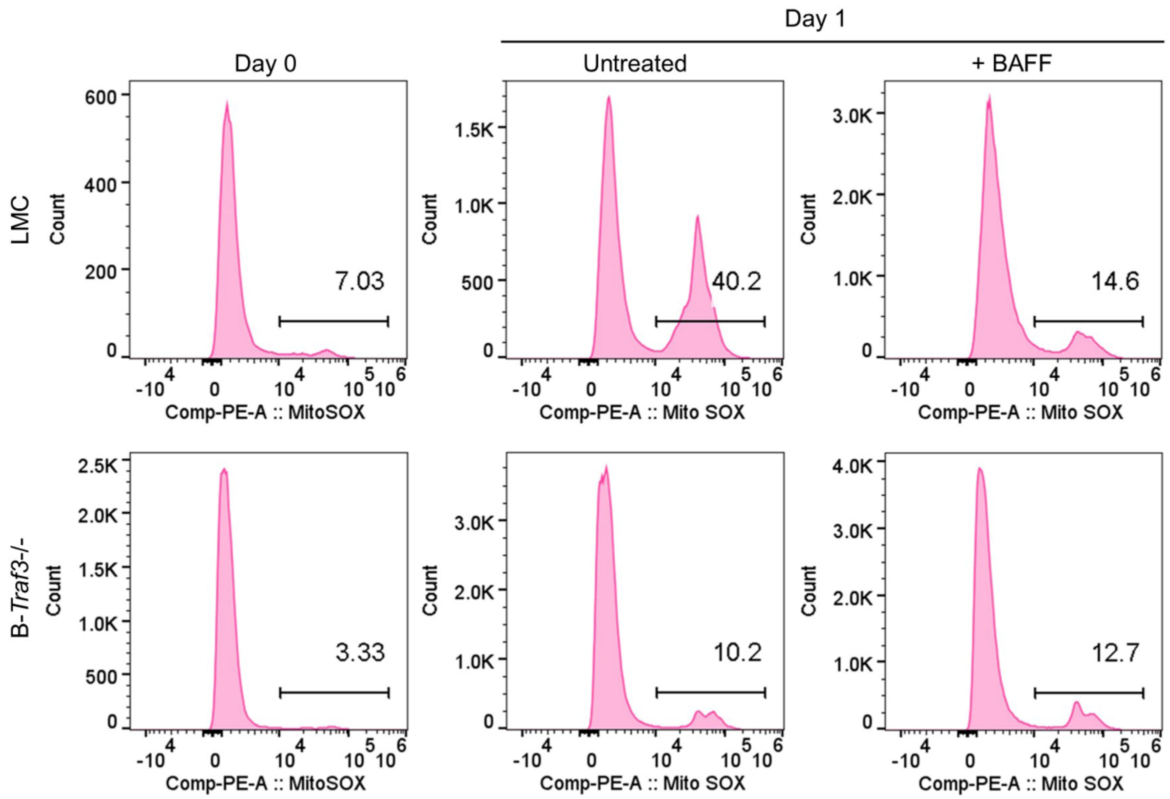

Supplementary Figure 7: BAFF treatment inhibited mitochondrial ROS production in LMC but not in *Traf3*$^{-/-}$ B cells. Splenic B cells were purified from gender-matched, young adult (8-12-week-old) naïve LMC or B-*Traf3*$^{-/-}$ mice. Purified cells were analyzed directly (Day 0) or at day 1 after *ex vivo* culture in mouse B cell medium, in the absence or presence of 0.5 µg/ml BAFF. Representative FACS profiles of MitoSOX Red staining are shown and gated populations indicate the MitoSOX Red^{hi} cells. FACS profiles shown are representative of 3 experiments.

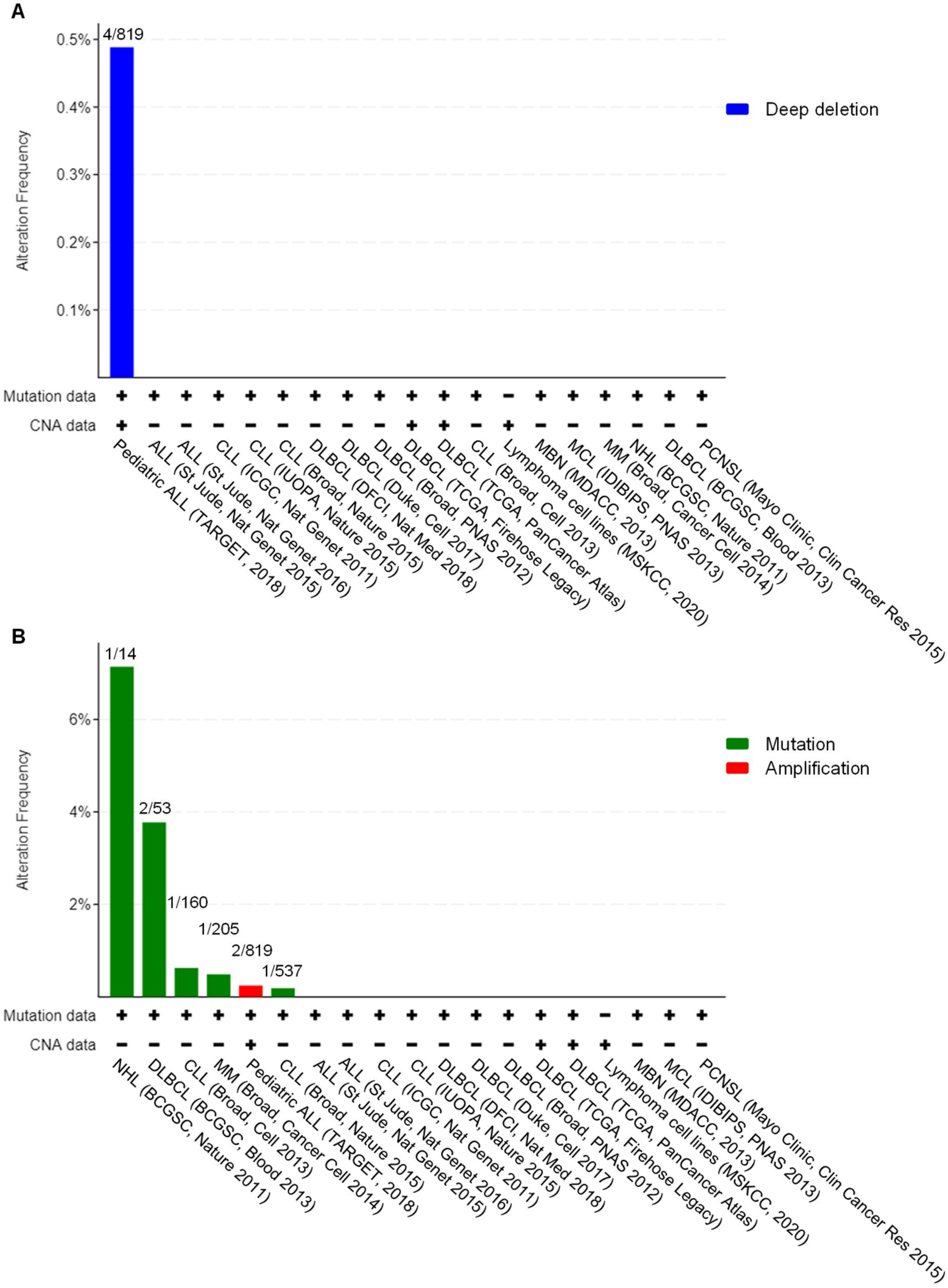

Supplementary Figure 8: **Genetic alterations of the *MFF* and *DNM1L* genes in human B cell malignancies.** Genetic alterations of *MFF* and *DNM1L* are retrieved from the TCGA database. Genetic alterations shown include deep deletion (copy number loss), mutation (missense or truncating mutation) and amplification (copy number gain).

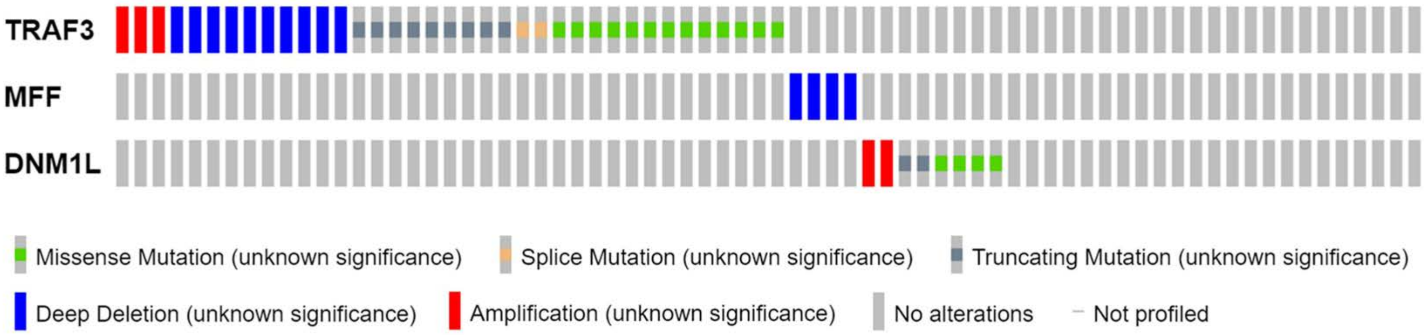

Supplementary Figure 9: **Combined genetic alterations of the *TRAF3, MFF* and *DNM1L* genes in human B cell malignancies.** Combined genetic alterations of *TRAF3*, *MFF* and *DNM1L* in human B cell malignancies were analyzed using the TCGA tool. The nature of the genetic alterations identified in each patient is indicated by a mutation symbol shown at the bottom legend of the figure.
